# Plasminogen activator inhibitor 1 is associated with high-grade serous ovarian cancer metastasis and is reduced in patients who have received neoadjuvant chemotherapy

**DOI:** 10.3389/fcell.2023.1150991

**Published:** 2023-12-07

**Authors:** Tanya E. Kelly, Cathy L. Spillane, Mark P. Ward, Karsten Hokamp, Yanmei Huang, Prerna Tewari, Cara M. Martin, Lucy A. Norris, Bashir M. Mohamed, Mark Bates, Robert Brooks, Stavros Selemidis, Douglas A. Brooks, Waseem Kamran, Feras Abu Saadeh, Sharon A. O’Toole, John J. O’Leary

**Affiliations:** ^1^ Department of Histopathology and Morbid Anatomy, School of Medicine, Trinity College Dublin, Dublin, Ireland; ^2^ Trinity St James’s Cancer Institute, St. James’s Hospital, Dublin, Ireland; ^3^ Smurfit Institute of Genetics, Trinity College Dublin, Dublin, Ireland; ^4^ School of Forensic Medicine, Xinxiang Medical University, Xinxiang, China; ^5^ Department of Obstetrics and Gynaecology, School of Medicine, Trinity College Dublin, Dublin, Ireland; ^6^ Sansom Institute for Health Research, University of South Australia, Adelaide, Australia; ^7^ School of Health and Biomedical Sciences, STEM College, Royal Melbourne Institute of Technology, Melbourne, VIC, Australia; ^8^ Division of Gynaegological Oncology, St. James’ Hospital, Dublin, Ireland

**Keywords:** ovarian, cancer, PAI-1, platelets, RNA-seq

## Abstract

**Introduction:** High-grade serous ovarian cancer (HGSOC) is the most prevalent and deadliest subtype of epithelial ovarian cancer (EOC), killing over 140,000 people annually. Morbidity and mortality are compounded by a lack of screening methods, and recurrence is common. Plasminogen-activator-inhibitor 1 (PAI-1, the protein product of SERPIN E1) is involved in hemostasis, extracellular matrix (ECM) remodeling, and tumor cell migration and invasion. Overexpression is associated with poor prognosis in EOC. Platelets significantly increase PAI-1 in cancer cells *in vitro*, and may contribute to the hematogenous metastasis of circulating tumor cells (CTCs). CTCs are viable tumor cells that intravasate and travel through the circulation–often aided by platelets - with the potential to form secondary metastases. Here, we provide evidence that PAI-1 is central to the platelet-cancer cell interactome, and plays a role in the metastatic cascade.

**Methods:** SK-OV-3 cells where PAI-1 had been silenced, treated with healthy donor platelets, and treated with platelet-conditioned medium were used as an *in vitro* model of metastatic EOC. Gene expression analysis was performed using RNA-Seq data from untreated cells and cells treated with PAI-1 siRNA or negative control, each with and without platelets. Four cohorts of banked patient plasma samples (n = 239) were assayed for PAI-1 by ELISA. Treatment-naïve (TN) whole blood (WB) samples were evaluated for CTCs in conjunction with PAI-1 evaluation in matched plasma.

**Results and discussion:** Significant phenotypic changes occurring when PAI-1 was silenced and when platelets were added to cells were reflected by RNA-seq data, with PAI-1 observed to be central to molecular mechanisms of EOC metastasis. Increased proliferation was observed in cells treated with platelets. Plasma PAI-1 significantly correlated with advanced disease in a TN cohort, and was significantly reduced in a neoadjuvant chemotherapy (NACT) cohort. PAI-1 demonstrated a trend towards significance in overall survival (OS) in the late-stage TN cohort, and correlation between PAI-1 and neutrophils in this cohort was significant. 72.7% (16/22) of TN patients with plasma PAI-1 levels higher than OS cutoff were CTC-positive. These data support a central role for PAI-1 in EOC metastasis, and highlight PAI-1’s potential as a biomarker, prognostic indicator, or gauge of treatment response in HGSOC.

## Introduction

Epithelial ovarian cancer (EOC) is the eighth leading cause of cancer death in women, and accounts for 85%–90% of all ovarian cancers. 75% are of the high-grade serous subtype (HGSOC). Lack of effective screening places 87% of diagnoses at stage III or IV. The most recent GloboCan study reports that cases are set to rise to 428,966 by 2040, with deaths set to increase to 313,617 (Sung et al., 2021). The relationship between platelets and cancer cells is integral to the hematogenous metastasis of many cancer types, including EOC. Thrombocytosis is frequently associated with advanced disease (Stone et al., 2012), and conversely, thrombocytopenia is associated with a lower risk of metastasis (Nieswandt et al., 1999). It has historically been thought that shedding of ovarian tumor cells into the circulation was rare, however, recent studies show that hematogenous metastasis of OC can arise (Pradeep et al., 2014). Additionally, Labelle demonstrated that platelet-cancer cell interactions induce epithelial-to-mesenchymal transition (EMT) and promote metastasis when intravasating CTCs are cloaked by platelets (Labelle et al., 2014). Platelet adhesion occurs across multiple cancer types, inducing a mesenchymal phenotype that amplifies metastatic potential (Spillane et al., 2021). Platelets are able to physically protect CTCs from shear stress in the circulation (Egan et al., 2014), also contributing to immune evasion (Placke et al., 2012), and CTCs themselves may hold the key to tumor genomes and proteomes (Ward et al., 2021). CTCs are viable tumor cells that intravasate at the primary tumor site and travel through the circulation. Platelet-cloaked CTCs travel through the blood, extravasating at secondary sites where, together with the platelets, they form metastatic niches (Labelle et al., 2014); however, CTCs are both rare and heterogeneous, making them difficult to detect, isolate, and culture (Barriere et al., 2014). Subpopulations include epithelial, epithelial-mesenchymal, mesenchymal, and mesenchymal-stem, and it is important to acknowledge that these subtypes exist on a gradient that may change over time rather than as discrete subsets (Barriere et al., 2014). Use of CTCs has been demonstrated as a prognostic marker in breast cancer (Cristofanilli et al., 2004) as well as other cancer types, including colorectal, gastric, lung, and prostate (Van Berckelaer et al., 2016). In EOC, smaller studies have yielded variable results regarding correlations between CTC enumeration, PFS, and OS, however larger studies have reported more encouraging results (Van Berckelaer et al., 2016). As there is no one specific marker expressed by all cancer cells, current detection is often achieved *via* size and surface marker exclusion. Adding to this difficulty is a backdrop of leukocytes that remains significant even after enrichment (Van Berckelaer et al., 2016). Identification by fluoroscopy involves the use of EpCAM, cytokeratins, N-cadherin, E-cadherin, and vimentin. CTC status has been shown to be positively associated with advanced tumor stage, and to positively correlate with CA-125 levels, post-surgical residual cancer, and shorter survival rates (Van Berckelaer et al., 2016). CTCs are currently being investigated as a tool in ovarian cancer screening with the goal of producing a diagnostic “liquid biopsy.” PAI-1 is a single-chain glycoprotein encoded by the serine protease inhibitor 1 (SERPIN E1) gene, composed of roughly 380 amino acids (aa) and having a molecular mass of approximately 50 kDa (kDa) (Declerck et al., 1992). A major inhibitor of fibrinolysis expressed by many different cell types, most notably endothelial cells and platelets, PAI-1 exists in 3 conformations: active, latent/inactive, and cleaved/substrate (Declerck et al., 1992). It is the main suppressor of tissue-type plasminogen activator (tPA) and urokinase-type plasminogen activator (uPA), and is regulated by many factors including growth factors, hormones, and cytokines (Declerck et al., 1992). PAI-1 is upregulated in conjunction with cellular plasticity in adults, most often during wound-healing (Wilkins-Port et al., 2010), regulating cell adhesion by binding to the somatomedin (SMB) domain of vitronectin (Deng et al., 2001) and also by inhibition, mediated by the urokinase-type plasminogen activator receptor (uPAR) (Czekay et al., 2011). It is an important molecule in cellular migration where, in concert with low-density lipoprotein receptor-related protein 1 (LPR1), vitronectin, and the uPA/uPAR complex, it assists in the modulation of requisite detachments and reattachments (Czekay et al., 2011). PAI-1 is found in the α-granules of platelets and is released upon activation. Platelets contain large amounts of transcriptionally-active PAI-1 messenger RNA (mRNA), and thus undergo continuous *de novo* synthesis of active PAI-1 (Brogren et al., 2004). Platelet-cancer cell interactions are capable of driving PAI-1 overexpression, and overexpression of PAI-1 in tumor cells and stroma has been implicated in many cancers, including breast, bladder, colon, cervix, kidney, prostate, skin, and ovary (Pappot et al., 1995). Supporting this, PAI-1 was previously identified by our group *via* Affymetrix arrays as one of the top five genes significantly dysregulated as a result of platelet-cancer cell interactions (Spillane et al., 2021). PAI-1 was altered in 90% of 16 cancer cell lines, spanning 7 cancer types, and including EOC (Egan et al., 2014). High levels of PAI-1 in tumor tissue extracts, serum, and plasma have been associated with poor prognosis in EOC (Chambers et al., 1998; Ho et al., 1999; Kuhn et al., 1999; Aune et al., 2012), and increased PAI-1 has been demonstrated to be mitogenic, pushing proliferating EOC cells through the G_1_ checkpoint, leaving mutations intact (Giacoia et al., 2014). It has been implicated in the peritoneal dissemination of metastatic OC cells (Peng et al., 2019), and elevated plasma PAI-1 levels were significantly associated with the presence of malignancy and late-stage OC disease (Ho et al., 1999). Transforming growth factor β (TGF-β) has been demonstrated to increase PAI-1 transcription (Dennler et al., 1998), however other mechanisms leading to increased PAI-1 and the routes by which PAI-1 contributes to metastasis have yet to be mapped. We provide evidence here of a central role for PAI-1 in the platelet-cancer cell interactome by interrogating the synergistic pressures that PAI-1 and platelets exert in both the tumor microenvironment and in the circulation, and by investigating how these may contribute to the differential gene expression of PAI-1 in the contexts of OC metastasis and patient prognosis.

## Materials and methods

***Cell Culture*** SK-OV-3 cells (ATCC) were cultured in McCoy’s 5A (Sigma) with 10% fetal bovine serum (FBS, Sigma), 2 mM L-Glutamine (Sigma), and 100U penicillin +0.1 mg streptomycin/mL (Sigma).

***Platelet Isolation from Donor Whole Blood*** Ethical approval for blood collection for all *in vitro* assays was granted on 24 June 2015 by the Coombe Women and Infants University Hospital (CWIUH) Ethics Committee: “Recruitment of Healthy Volunteers for *in vitro* Studies in the Metastatic Cascade”. Healthy donors who had not taken medications known to affect platelet function for ≥10 days were recruited, and platelets were isolated from consented donor whole blood. Blood was collected in 10% acid citrate dextrose (ACD), rocked at 30 revolutions per minute (RPM) for 15 min (min), and then aliquoted and centrifuged at 170 relative centrifugal force (RCF) for 10 min at room temperature (RT). The upper layer of platelet-rich plasma (PRP) was removed and acidified to pH 6.5 with ACD. Prostaglandin 2 (PGE2, Sigma) was added to a final concentration of 1 μM. PRP was centrifuged at 720 RCF for 10 min at RT, the supernatant removed, and the platelet pellet was resuspended in a modified Tyrode’s Buffer (JNL buffer). Platelets were counted using the Sysmex XP-300 (Sysmex Europe, GmbH), and activated prior to use with 0.18 mM calcium chloride (CaCl_2_). Activation prior to addition of CaCl_2_ was ruled out by flow cytometry (Additional File 1) using the FACS-Melody (Becton-Dickinson, United States).

***Silencing of PAI-1 with siRNA*** PAI-1 was silenced *via* siRNA (Ambion Silencer Select SERPIN E1 siRNA) in an RNAi-MAX Lipofectamine (Thermo-Fisher) complex (LCS) according to the manufacturer’s instructions. This particular siRNA had previously been optimized by our group and was found to be the most appropriate. Cells received 10 nM PAI-1 siRNA in LCS, 10 nM negative control (Ambion Silencer Select ss#2) in LCS, or were left untreated. At 24 h, platelets were added to cells at 1,000:1. After another 24 h incubation, the cell monolayer was dissociated with Trypsin-EDTA (Sigma), cells were washed in Dulbecco’s phosphate-buffered saline (DPBS) and pelleted for RNA extraction.

***RNA Extraction and Real-time Quantitative PCR*** (***RT-qPCR***) RNA was extracted using Qiagen’s RNeasy MiniSpin Kit, quantitation performed using Invitrogen’s Qubit 3.0 fluorometer with Invitrogen’s Qubit RNA HS Assay Kit, and copy deoxyribonucleic acid (cDNA) synthesized from 100 ng total RNA using Applied Bioscience’s High-Capacity Reverse Transcription cDNA kit, all per manufacturer’s instructions. Real-time quantitative polymerase chain reaction (RT-qPCR) was performed on Applied Biosystems’ AB7900, using Applied Biosystems’ TaqMan Gene Expression Assay for SERPIN E1 (Hs01126606_m1; Applied Biosystems, Lot #P170504-007 B03), with GAPDH as control (Hu GAPDH FAM MGB; Applied Biosystems, Lot# 4352934-1301,038). Samples were run in triplicate, and mRNA transcripts quantitated by the 2^−ΔΔCt^ method (Livak KJ, Schmittgen TD. “Analysis of relative gene expression data using real-time quantitative PCR and the 2^−ΔΔCT^ Method. Methods.” 2001; 25 (4):402-408. doi:10.1006/meth. 2001.1262). RT-qPCR results were log10 transformed and analyzed in GraphPad Prism using a one-way analysis of variance (ANOVA) with Tukey’s *post hoc* test.

***Enzyme-linked Immunosorbent Assay*** (***ELISA***) Protein extraction from cell line samples was performed using mammalian protein extraction reagent (MPER; Sigma, United States), with quantitation performed using Pierce’s bicinchoninic acid assay (BCA, Thermo Fisher, United States). Cell line PAI-1 protein concentrations were determined using the Zymutest PAI-1 ELISA detection kit (Hyphen-Biomed, France) according to the manufacturer’s instructions. Undiluted patient plasma samples were assayed using R&D’s Human SERPIN E1/PAI-1 DuoSet (R&D Systems, Minnesota, United States) and its ancillary kit according to the manufacturer’s instructions.

***Wound-healing Assay*** Cells were seeded at a concentration of 10^5^ per well in 9 wells of a 12-well tissue culture plate and incubated overnight. Medium was then replaced with serum-free antibiotic-free medium. Healthy donor platelets were isolated from whole blood as described above. 1,000:1 platelets were added to cells (Cooke et al., 2015) in 6 of the wells labeled platelets/fresh medium (PLT/FM) and platelets/releasate (PLT/REL) and incubated for 24 h. Medium was aspirated, cells were washed with DPBS, and a scratch made in each well with a pipette tip. Untreated cells (UNT) and cells in the well labeled PLT/FM were given fresh medium. Wells labeled PLT/REL received platelet releasate. Platelet releasate (platelet-conditioned medium) was obtained by aspirating the medium from those wells where cells had been treated with platelets. This medium was centrifuged to remove particulate, and was then added back to the wells from which it had initially been aspirated. Cells were incubated overnight, and photographed at 0, 6, 20, and 24 h. Images were evaluated for wound closure with ImageJ (Public Domain, NIH, United States). Wound edge migration velocity was determined by the equation average velocity equal to the change in distance divided by the change in time ( $\bar{\nu }=\Delta x/\Delta t$ ) and acceleration determined by the equation average acceleration equal to the change in velocity divided by the change in time ( $\bar{a}=\Delta \upsilon /\Delta t$ ). Area values in millimeters squared (mm^2^) were converted to distance = x mm by taking the quotients of those areas when they were divided by the constant *y*-axis values in mm. Results were multiplied by 1,000 to give $\bar{v}$ in micrometers per hour (μm/h), and $\bar{a}$ in micrometers per hour per hour (μm/h/h), or micrometers per hour squared (μm/h^2^).

***PAI-1 Silencing/Wound-healing Assay*** A wound-healing assay which incorporated silencing of PAI-1 was performed. This was achieved by combining the aforementioned protocols for the wound-healing and silencing of PAI-1 with siRNA assays. Cells were seeded in the wells of two tissue culture plates, each labeled with FM (fresh medium), PLT/FM (platelets and fresh medium), or PLT/REL (platelets and platelet releasate) at a density of 10^5^ cells per well and incubated overnight to allow adherence. PAI-1 was silenced *via* siRNA (Ambion Silencer Select SERPIN E1 siRNA) in an RNAi-MAX Lipofectamine (Thermo-Fisher) complex (LCS) according to the manufacturer’s instructions. Cells in one plate received 10 nM PAI-1 siRNA in LCS and in the other received 10 nM negative control (Ambion Silencer Select ss#2) in LCS. Both the siRNA and the negative control had previously been optimized by our group. At 24 h, platelets were added to cells in each plate in wells labeled PLT/FM and PLT/REL at 1,000:1. Wells labeled FM received no platelets. After another 24 h, the medium was aspirated from all wells, cells were washed with DPBS, and a scratch was made in each well with a pipette tip. At this stage, cells labeled FM and PLT/FM were given fresh medium. Wells labeled PLT/REL received platelet releasate, which is platelet-conditioned medium obtained by aspirating the medium from those wells where cells had been treated with platelets. This platelet-conditioned medium was centrifuged to remove particulate and cell debris, and was then added back to the wells from which it had initially been aspirated. Cells were incubated overnight, and photographed at 0, 6, 20, and 24 h. Images were evaluated for wound closure with ImageJ (Public Domain, NIH, United States).

***Invasion Assay*** SK-OV-3 cells were grown in McCoy’s 5A supplemented with 10% FBS, 2 mM L-Glutamine, and 100 U penicillin +0.1 mg streptomycin/mL. Cells were washed, trypsinised, and counted, and 4.0 × 10^4^ cells were seeded in serum-free and antibiotic-free McCoy’s 5A in the wells of a 12-well plate and allowed to grow for 24 h. Per the manufacturer’s protocol, cells were synchronized by serum-starvation for 24 h prior to seeding in the invasion assay chambers. For the PAI-1 knockdown: medium was aspirated from all wells, and cells washed in DPBS. 800 μL fresh medium were added to all wells, and transformation with PAI-1 siRNA was performed with untreated cells (UNT) receiving 200 μL Opti-MEM, siRNA (siRNA) wells receiving 200 μL of 50 nM PAI-1 siRNA/Lipofectamine complex (10 nM final concentration in wells), and siNEG wells (CTL) receiving 200 μL of 50 nM siNEG/Lipofectamine complex (10 nM final concentration in wells). For assays receiving platelets, platelets were isolated from whole donor blood as previously described and added at a ratio of 1,000:1 at 24 h post-transformation. The invasion assay chambers were seeded after another 24 h. Polyethylene terephthalate (PET) membrane transwell inserts with 8.0 μm pores were coated with Engelbreth-Holm-Swarm (EHS) mouse sarcoma cell-secreted extracellular matrix (ECM) protein per the Corning^®^ Matrigel^®^ cell invasion assay protocol. 25 μL of previously aliquoted ECM gel (stock concentration = 9.72 mg/mL) were thawed on ice and added to 975 μL of ice-cold Tris-NaCl coating buffer per the manufacturer’s protocol for a final concentration of 250 μg/mL 100 μL of this solution were pipetted into each of 9 8.0 μM PET cell culture inserts, and incubated at 37°C for 2 h. After 2 h, the remaining liquid was carefully pipetted off, and 700 μL of McCoy’s 5A containing 5% FBS as chemoattractant were added to the lower chambers. 2.5 × 10^4^ cells were added in serum-free medium to individual wells labelled according to their treatment type, with a full set of matching controls (no ECM gel). These were returned to the incubator and allowed to incubate overnight. Cells were removed from the apical side of the inserts using unsupplemented McCoy’s 5A and cotton swabs. Cells remaining on the basolateral sides of the inserts were fixed in ice-cold 100% methanol (Acros Organics, United States) for 2 h. Crystal violet stain was made by dissolving 0.125 g crystal violet in 50 mL of 20% methanol. Cells were counted manually by microscopy photography according to the manufacturer’s protocol. This was accomplished by photographing 5 random fields per insert *via* the ocular lens, manually counting cells per field, and then taking the average of those counts. Invasion assay results were analyzed in GraphPad Prism without transformation.

***RNA-Sequencing*** (***RNA-Seq***) Three independent sets of RNA were chosen for analysis by RNASeq based on RT-qPCR assays, two of which were matched with invasion assays. Culturing of SK-OV-3 cells, platelet isolation, and platelet:cell ratio was performed as described above. RT-qPCR and invasion assays were performed as described above, each using independent cell passages and independent platelet isolations. Each set of RNA samples was composed of untreated (UNT), treated with a negative control siRNA (siNEG), treated with PAI-1 siRNA (siRNA), untreated with platelets (UNT + P), siNEG with platelets (siNEG + P), and PAI-1 siRNA with platelets (siRNA + P). RNA was quantitated by Qubit 3 fluorimeter (Invitrogen, United States). RNA integrity was determined by Bioanalyzer 2,100 (Agilent). Poly-A tail capture and ligation-based adaptor addition was employed to target the coding transcriptome using the TruSeq Stranded mRNA Library Prep Kit (Illumina Inc., San Diego, CA, United States). A library pool was constructed and 2 × 75 bp paired-end (PE) reads were obtained using the NovaSeq6000 (Illumina, Inc., United States). Sample read quality was checked using FastQC (Babraham Bioinformatics, United Kingdom). Samples were checked for contamination against human, mouse, rat, and plasmid index genomes using FastQ-Screen (Babraham Bioinformatics, United Kingdom) in conjunction with the Bowtie2 sequence alignment tool (Center for Computational Biology, Whiting School of Engineering, Johns Hopkins University, Baltimore, MD, United States). Sequences were trimmed and corrected for potential batch effect using the ComBat-Seq negative binomial method (Zhang, JD, Ruschaupt M, and Biczok R. “ddCt Method for qRT-PCR data analysis.” Open-source Bioconductor software package. 2017.). Resulting transcript counts are included as Additional File 2. Analyses were performed using online resources, including Integrative Genomics Viewer (IGV, The Broad Institute, Cambridge, MA United States), iDEP92, ShinyGO and GSEA (Bioinformatics, South Dakota State University, SD, United States), and Search Tool for the Retrieval of Interacting proteins (STRING Consortium). *p*-values were adjusted after Benjamini–Hochberg correction. Significance criteria were an adjusted *p*-value less than or equal to 0.05, a minimum expression-fold change of 1.5, and a false discovery rate (FDR) of 0.05 or less.

***Plasma PAI-1 Evaluation in Patient Cohorts by ELISA*** Ethical approval for the samples used in the retrospective study was granted as part of the gynaecological biobank on 8 December 2004 by the St. James’ Hospital/Tallaght University Hospital Joint Research Ethics Committee: “Gynaecological Cancer Bioresource”, Project ID 2095. Plasma samples from patients with HGSOC diagnoses or benign serous histology between 2011 and 2019 were included in this study if informed consent had been received to have samples stored in the DISCOVARY biobank for research purposes. 4.5 mL of whole blood were collected in sodium citrate and were processed and stored within 1 h. Samples were centrifuged at 4°C for 20 min at 2000 RCF. Platelet-free plasma (PFP) was removed, aliquoted, snap frozen in liquid nitrogen, and stored at −80°C until assay. Samples were placed into one of four cohorts based on histology: treatment-naïve (TN, n = 110), undergoing cytoreductive surgery post neoadjuvant chemotherapy (NACT, n = 79), recurrent disease (n = 15), and benign serous histology (n = 35). The occurrence of a venous thrombotic event (VTE) was the sole exclusion criterion. Available biometric, hematological, and biochemical data were compiled and stratified by disease stage, and it should be noted that not all data were available for all patients. All patients in the NACT had late-stage (stage III or IV) disease at the time of clinical diagnosis. 10 patients from this cohort had a positive response to chemotherapy resulting in a pathological stage of I or II. These patients were excluded from comparisons where having late-stage disease was a criterion. Plasma PAI-1 was evaluated by ELISA (R&D) as described above.

***CTC Enumeration from Whole Blood Samples*** Ethical approval for the use of ovarian cancer patient samples for prospective circulating tumour cell (CTC) isolation and enumeration was granted on 4 December 2017 by the St. James’ Hospital/Tallaght University Hospital Joint Research Ethics Committee: “Deciphering the most clinically and biologically relevant circulating tumour cells [CTCs] in cancer metastasis”, Project ID 44. Written, informed consent was obtained from all volunteer blood donors and ovarian cancer patients prior to participation. 7.5 mL of whole blood (WB) were collected prospectively in anticoagulant ethylenediaminetetraacetic acid (EDTA) from late-stage HGSOC treatment-naïve (TN) patients presenting for primary surgery or just prior to neoadjuvant chemotherapy (NACT) administration. CTCs were isolated from WB using the Parsortix^®^ (Angle plc) cell separation system according to the manufacturer’s protocol, stained, and counted based on EpCAM and pan-cytokeratin positivity *via* fluorescence microscopy.

***Statistical Analysis*** GraphPad Prism (San Diego, CA, United States) was used to perform all statistics, except for RNA-Seq analytics which employ their own integrated statistical analyses, and overall survival (OS), which was calculated in SPSS using a Kaplan-Meier curve. Log10 transformations were used for those analyses where data were not normally distributed, and a Kruskal–Wallis non-parametric ANOVA was used for those data that continued to demonstrate non-normal residuals post transformation. OS was calculated using PAI-1 as the factor, and an R-based ROC curve method was used to determine the survival cutoff point. For all tests, a *p*-value less than or equal to 0.05 was considered significant.

## Results

**FIGURE 1:**
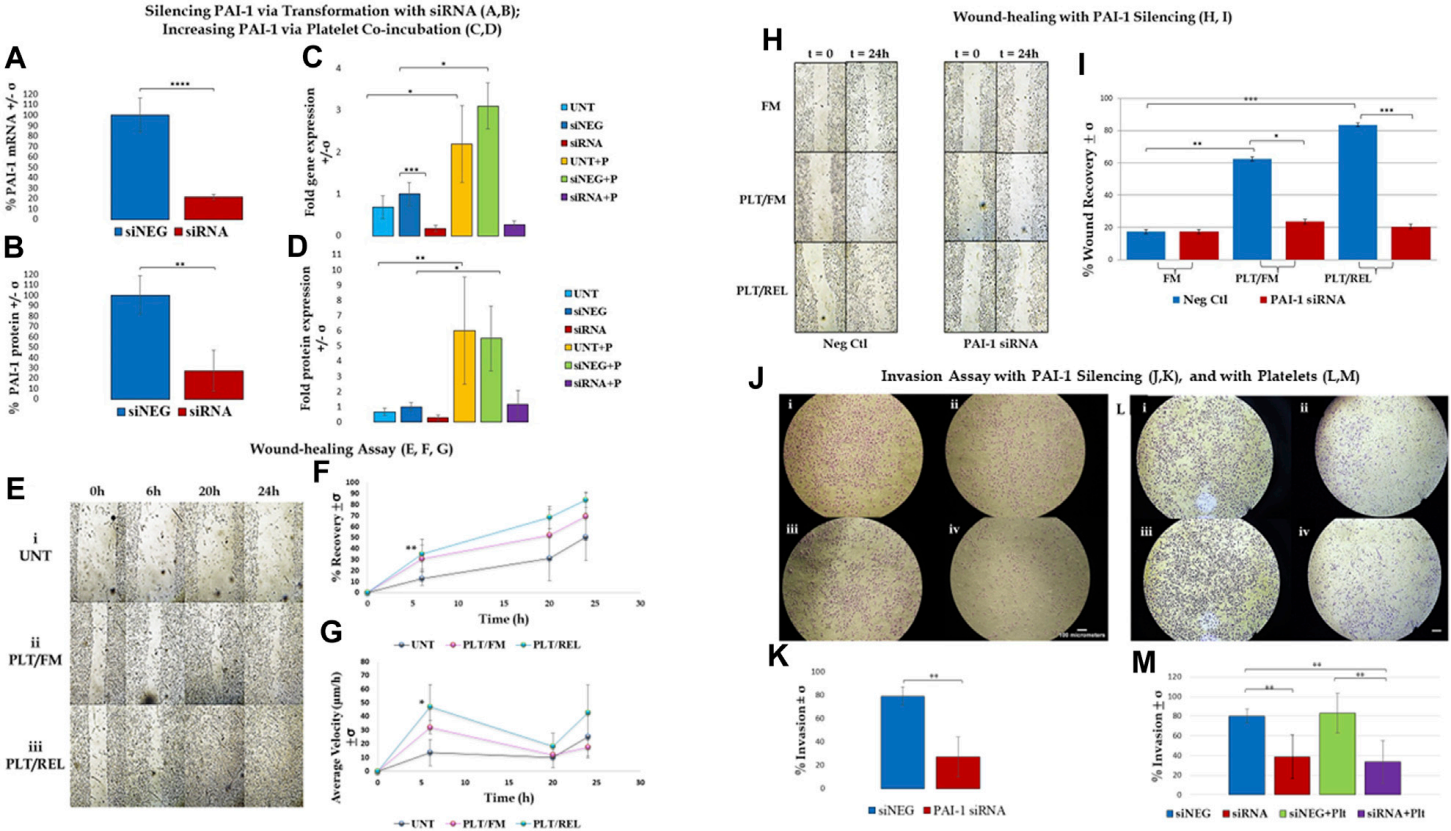
Assay results are shown +/- standard deviation (σ) and demonstrate that PAI-1 is integral to a metastatic phenotype, and that platelet-cancer cell interactions increase PAI-1 and contribute to this phenotype. **(A,B)**: Silencing PAI-1 *via* transformation with siRNA; **(A)**. RT-qPCR results show that PAI-1 mRNA is reduced when SK-OV-3 cells are treated with siRNA (red) compared to negative control (blue) (2-tailed *t*-test, n = 3, *p* ≤ 0.0001); **(B).** ELISA results show that PAI-1 protein is reduced when cells are treated with siRNA (red) compared to negative control (blue) (2-tailed *t*-test, n = 3, *p* = 0.0069). **(C,D)**: Increasing PAI-1 *via* platelet co-incubation; **(C)**. One-way ANOVA (n = 3) of untreated (light blue), negative control siNEG (darker blue), PAI-1 siRNA (red), untreated plus platelets (yellow), negative control plus platelets (green), and PAI-1 siRNA plus platelets (purple) was significant (*p* ≤ 0.0001). Platelet coincubation with untreated cells yielded a 3.2-fold increase in PAI-1 mRNA *versus* untreated alone (*p* = 0.0143), and there was a 3.1-fold increase in PAI-1 mRNA in the negative control with platelets (*p* = 0.0124) *versus* cells treated with negative control alone. **(D).** The same treatment types mentioned in Panel C (color key is the same) were tested by ELISA for PAI-1 protein. A one-way ANOVA was significant (n = 3, *p* = 0.0001). PAI-1 protein expression was increased when platelets were added to both untreated cells (9.1-fold, *p* = 0.0071) and to cells treated with a negative control (5.5-fold, *p* = 0.0327). **(E–G)**: Wound-healing assay; Wound-healing is increased in SK-OV-3 cells by co-incubation with platelets and releasate (n = 3): **(E).** Photographs taken at 0, 6, 20, and 24 h: (**i)** untreated cells, (**ii)** cells treated with platelets and fresh medium, (**iii)** cells treated with platelets and releasate; **(F).** Percent wound closure from 0 to 6 h is greater in cells treated with both platelets and releasate (teal line 84.4%) compared to untreated cells (grey line 50.6%), with *p* = 0.0096. **(G).** Wound edge velocity is significant between cells treated with platelets/releasate (teal line, 47.1 μm/h) and untreated cells (grey line, 13.6 μm/h) from 0–6 h (*p* = 0.0388). **(H,I)**: Wound-healing with PAI-1 silencing; **(H)**. Photographs taken at 0 and 24 h of SK-OV-3 cells treated with a negative control or PAI-1 siRNA that were untreated (UNT), treated with platelets and then fresh medium (PLT/FM), or treated with platelets and platelet releasate (PLT/REL). **(I)**. Wound-healing assay (n = 3) demonstrates that wound healing in cells treated with a negative control (blue bars) is increased when cells are co-incubated with platelets (49.96%, *p* = 0.0041) and platelets and releasate (86.9%, *p* = 0.0003) *versus* no platelets (17.4%). Cells treated with PAI-1 siRNA (red bars) are not significantly affected by co-incubation with either platelets or platelets and releasate. Wound closure is inhibited by PAI-1 siRNA in the presence of platelets and their releasate compared to negative control (*p* = 0.0005). **(J,K)**: Invasion assay with PAI-1 silencing; **(J)**. **(i)** SK-OV-3 cells treated with negative control siRNA invaded through a control membrane, (**ii)** cells treated with PAI-1 siRNA through a control membrane, **(iii)** 79% of cells treated with negative control siRNA invaded through an ECM gel-coated membrane, (**iv)** 27% of cells treated with PAI-1 siRNA invaded through an ECM gel-coated membrane, **(K).** Invasion assay demonstrated a 52% loss of invasion when PAI-1was silenced (red) vs negative control (blue) (2-tailed *t*-test, n = 4, *p* = 0.008). **(L,M)**: Invasion assay with platelets; **(L)**. (**i)** 79.9% of SK-OV-3 cells treated with negative control invaded through ECM-coated PET membrane *versus* **(ii)** 38.5% of cells treated with PAI-1 siRNA, (**iii**) 83% of cells treated with negative control and platelets invaded through ECM-coated PET membrane *versus* **(iv)** 33.7% of cells treated with PAI-1 siRNA and platelets; **(M)**. Silencing PAI-1 (red) significantly decreased invasion by more than 40%, even in the presence of platelets (purple) (2-tailed *t*-test, n = 6, *p* = 0.0033), and platelets increased invasion in cells treated with a negative control (green) compared to negative control alone (blue).*p ≤ 0.05, **p ≤ 0.01, ***p ≤ 0.001, ****p ≤ 0.0001.

***PAI-1 and Platelets drive a Metastatic Phenotype in Ovarian Cancer Cells*** RT-qPCR revealed an average 78.4% loss of PAI-1 mRNA transcripts in SK-OV-3 cells treated with siRNA *versus* negative control (siNEG) (Figure 1A; n = 3, *p* ≤ 0.0001). This was further validated by a 73.3% loss of PAI-1 protein (Figure 1B; n = 3, *p* = 0.0069) in siRNA-treated cells *versus* siNEG. A second set of RT-qPCR assays (n = 3) in which SK-OV-3 cells were untreated, treated with a negative control siNEG, treated with PAI-1 siRNA, untreated plus platelets, siNEG plus platelets, and PAI-1 siRNA plus platelets was significant by one-way ANOVA with *p* ≤ 0.0001, and Tukey’s *post hoc* significance demonstrated that the addition of platelets to SK-OV-3 cells treated with negative control siNEG yielded a fold increase of 3.1 ± 0.55 in mRNA transcription (Figure 1C; n = 3, *p* = 0.0143) when compared to cells treated with negative control alone. Similarly, the addition of platelets to untreated cells yielded a fold increase of 3.2 ± 0.27 in PAI-1 mRNA transcripts, with *p* = 0.0124. No significant difference was noted in mRNA transcripts in cells treated with PAI-1 siRNA and cells treated with PAI-1 siRNA that were also treated with platelets (*p* = 0.6728). There was no significant difference in mRNA transcripts between the negative control and untreated cells when treated with platelets (*p* = 0.7928). A one-way ANOVA of the aforementioned treatment types were tested by ELISA for PAI-1 protein, with *p* = 0.0001. Tukey’s *post hoc* test demonstrated the significance of a 5.5-fold increase in PAI-1 protein expression when platelets were added to cells treated with a negative control (Figure 1D, n = 3, *p* = 0.0327) and a 9.1-fold increase when platelets were added to untreated cells (*p* = 0.0071). Both PAI-1 mRNA transcripts and protein were increased by the addition of platelets when PAI-1 was transiently silenced, and it is noted that the addition of platelets was not able to recover PAI-1 mRNA or protein to its pre-knockdown status (Figures 1C,D). Previous research in our lab - in which the same number of healthy donor platelets used for *in vitro* assays underwent RNA extraction and subsequent RT-qPCR - demonstrated that healthy donor platelets contained no detectable PAI-1 mRNA. The effect of platelets and their releasate on the motility of SK-OV-3 cells was assessed by wound-healing assay. Average percent wound closures were 50.6% in untreated cells (UNT, Figs. 1Ei and 1F (grey line)), 69.4% in cells treated with platelets/fresh medium (PLT/FM, Figs. 1Eii and 1 F (pink line)), and 84.4% in cells treated with platelets/releasate (PLT/REL, Figs. 1Eiii and 1F (teal line)). Wound closure rates are reported as millimeters squared per hour (mm^2^/h) in Table 1. A one-way ANOVA of wound closure rates was significant (n = 3, *p* = 0.0118), with Tukey’s *post hoc* significance revealing that exposure to platelets and their releasate increased the rate of wound closure significantly from 0 to 6 h, with *p* = 0.0096 (Figures 1E,F). Tables 2 and 3 list the respective velocities and accelerations of wound edge migration by treatment type. Velocity is equal to a change in position in a particular direction over time ( $\bar{v}$ = Δx/Δt) and is reported as micrometers per hour (μm/h). Acceleration is equal to the change in velocity over time ( $\bar{a}$ = Δ V /Δt) and is reported as micrometers per hour per hour or micrometers per hour squared (μm/h^2^). A one-way ANOVA of velocities was significant (n = 3, *p* = 0.0054), with Tukey’s *post hoc* significance between untreated cells and cells treated with platelets/releasate at 6 h (*p* = 0.0388). Figure 1G represents wound-edge accelerations, plotted as change in velocity (*Δ* $\left.\upsilon \right)$ over change in time (Δt), with deceleration–also demonstrated in Tables 1–3 - evident in all treatment types between 6 h and 20 h.

**TABLE 1:** Wound recovery rate in millimeters squared per hour (mm^2^/h). **Exposure to platelets and their releasate increased the rate of wound closure significantly compared to untreated cells between 0 h and 6 h (n = 3, *p* = 0.0096). UNT = untreated, PLT/FM = platelets and fresh medium, PLT/REL = platelets and releasate.

Wound recovery rate (mm^2^/h)			
Elapsed Time(h)	UNT	PLT/FM	PLT/REL
0–6	0.94758	4.58466	6.90616**
6–20	0.66561	1.23565	2.14428
20–24	4.80021	3.68068	2.75759

**TABLE 2:** Average velocity: $\bar{v}$ = Δx/Δt = rate of edge migration in micrometers per hour: Untreated cells move faster during the 20–24 h period, while cells treated with platelets/fresh medium or platelets/releasate move faster during the 0–6 h period. *Velocity was significantly increased in cells treated with platelets and releasate *versus* untreated cells between 0 h and 6 h (n = 3, *p* = 0.0388).

Average velocities of wound edge migration (μm/h)			
Elapsed time (h)	UNT	PLT/FM	PLT/REL
0–6	13.5888	32.0836	47.1435975*
6–20	10.1263	11.7729	18.26118987
20–24	25.3892	17.6229	42.97097295

**TABLE 3:** Average accelerations of wound edge migration in micrometers per hour per hour (μm/h^2^) $\bar{a}$ = Δ V /Δt., n = 3. Untreated cells experience greater acceleration during the period from 20–24 h, while cells treated with platelets/fresh medium or platelets/releasate experience greater acceleration during the period from 0–6 h. Cells from all treatment types experience deceleration during the time period from 6–20 h.

Average accelerations of wound edge migration (μm/h^2^)			
Elapsed time (h)	UNT	PLT/FM	PLT/REL
0–6	0.88115	3.374414139	6.472150029
6–20	−0.1845	−0.931529986	−2.008744026
20–24	1.74498	2.272492254	4.641850481

Cell-cycle analysis (Additional File 3) showed an increase in the number of cells treated with platelets and releasate that were in G_2_ at 20 h *versus* untreated cells and cells treated with platelets alone. This was inversely proportional to the rate of wound closure demonstrated in Figure 1G.

Wound healing was significantly inhibited in SK-OV-3 cells treated with platelets and their releasate (PLT/REL) when PAI-1 was silenced compared to cells treated with platelets and their releasate and a negative control siRNA (Figures 1H,I, n = 3, *p* = 0.0005). Average percent wound recovery was 17.4% in cells treated with negative control and fresh medium, 49.96% in cells treated with negative control and platelets/fresh medium, and 86.9% in cells treated with negative control and platelets/releasate. Coincubation with platelets and fresh medium (PLT/FM) significantly expedited wound-healing in cells treated with a negative control compared to cells treated with a negative control and fresh medium (FM) alone (Figures 1H,I, n = 3, *p* = 0.0041). This was significantly enhanced when cells were further incubated with platelet releasate. (Figures 1H,I, n = 3, *p* = 0.0003).

Invasion was significantly decreased from 79% to 27% (Figures 1J,K, n = 4, *p* = 0.0014) when cells were treated with PAI-1 siRNA for 48 h as compared to cells treated with a negative control. In a second set of invasion assays investigating the effect of platelets on invasion, a one-way ANOVA was significant with *p* = 0.0002, with a significant Tukey’s *post hoc* test where SK-OV-3 cells treated with PAI-1 siRNA were observed to be 45% less invasive than cells treated with a negative control (Figures 1L,M, n = 6, *p* = 0.0033). The addition of platelets increased invasion in both treatment groups. Importantly, the addition of platelets to cells where PAI-1 had been silenced did not recover the original invasive phenotype. This was demonstrated by a significant difference in invasion between cells treated with a negative control and those treated with both PAI-1 siRNA and platelets (n = 6, *p* = 0.002), and mirrors results observed in RT-qPCR and protein assays.

IF staining (Additional File 4) demonstrates increased PAI-1 in both untreated and siNEG-treated cells, and a visible loss of intracellular PAI-1 in our knockdown model. Consistent with RT-qPCR results, adding platelets to the knockdown model did not show increased PAI-1 staining.

**FIGURE 2:**
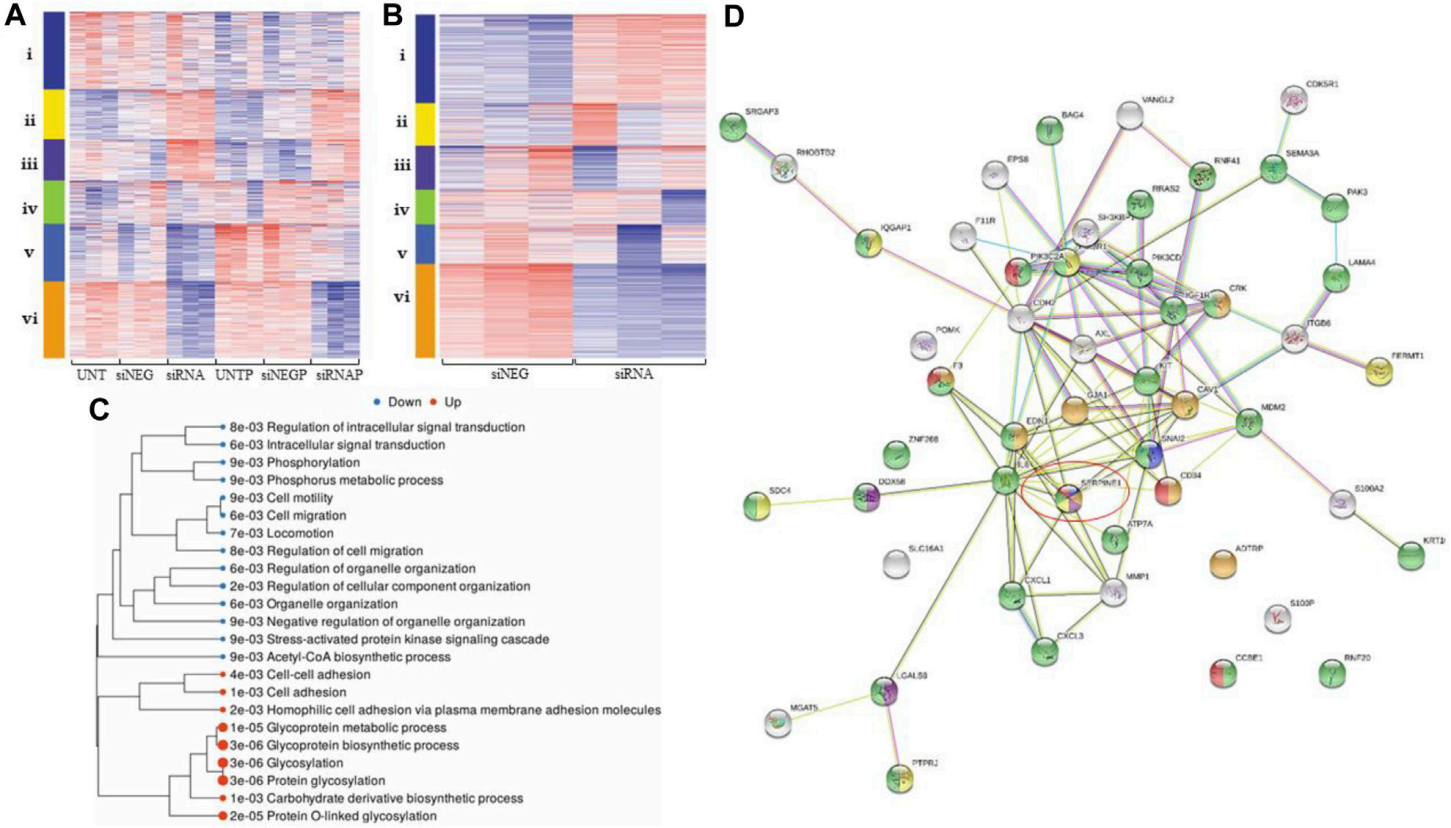
Differential gene expression analysis demonstrates that loss of PAI-1 disrupts pathways integral to metastatic processes. For all heatmaps, up- and downregulation are represented by red and blue, respectively. For all treatment type, n = 3. Sequences were trimmed and corrected for potential batch effect using the ComBat-Seq negative binomial method. Analyses were performed using online resources, including Integrative Genomics Viewer (IGV, The Broad Institute, Cambridge, MA United States), iDEP92, ShinyGO and GSEA (Bioinformatics, South Dakota State University, SD, United States), and Search Tool for the Retrieval of Interacting proteins (STRING Consortium). *p*-values were adjusted after Benjamini–Hochberg correction. Significance criteria were an adjusted *p*-value less than or equal to 0.05, a minimum expression-fold change of 1.5, and a false discovery rate (FDR) of 0.05 or less. **(A)**. Cluster analysis in iDEP92 demonstrates up- and downregulation of gene expression among treatment types including: (**i)** biosynthetic processes of sterols, cholesterols, and lipids, (**ii)** acute-phase response, leukocyte migration, (**iii)** glycosylation, (**iv)** cytokine stimulation, immune response, (**v)** cell migration, angiogenesis, and (**vi)** response to virus and type 1 interferon signaling; **(B)**. Cluster analysis demonstrates differential gene expression between PAI-1 knockdown (siRNA) and negative control (siNEG), including **i** glycosylation, **ii** cholesterol and sterol biosynthesis and metabolism, **iii** response to lipids and lipopolysaccharides, **iv** response to jasmonic acid, **v** response to virus, innate immune response, and **vi** cell migration and Acetyl-CoA biosynthesis from pyruvic acid; **(C)**. Tree of significantly enriched pathways with larger dots representing smaller *p*-values; **(D)**. gene map generated by STRING demonstrating that SERPIN E1 (circled) is central to ovarian cancer metastatic processes: negative regulation of cell adhesion mediated by integrin (blue), regulation of wound healing (orange), positive regulation of interleukin-8 (IL-8) production (purple), regulation of cell-matrix adhesion (yellow), regulation of cell migration (green), and positive regulation of angiogenesis (red).

***PAI-1 and Platelets augment Gene Expression in Metastatic Pathways in Ovarian Cancer Cells*** A cluster analysis (Figure 2A) demonstrated differentially-expressed genes (DEGs), with downregulation (blue) and upregulation (red) of genes corresponding to treatment types: untreated cells (UNT), cells treated with a negative control (siNEG), cells where PAI-1 had been silenced (siRNA), platelets added to untreated cells (UNT + P), platelets added to cells treated with a negative control (siNEG + P), or platelets added to cells where PAI-1 had been silenced (siRNA + P). Cluster i was composed of 224 genes involved in the biosynthetic processes of sterols, cholesterols, and lipids as well as their metabolic processes. 144 dysregulated genes in cluster ii were those involved with acute-phase response, leukocyte migration, and inflammatory response, and 119 dysregulated genes in cluster iii were described as those involved in the processes of protein glycosylation, glycoprotein biosynthesis, and transsynaptic signaling by soluble gases. 125 variable genes comprised cluster iv. These mainly showed an upregulatory response to the addition of platelets to our untreated and negative control samples, and were those involved in response to bacterial infection, cytokine stimulation, cytokine-mediated signaling, cellular response to oxygen-containing compounds, and immune response. Cluster v was composed of 166 variable genes that were highly downregulated in response to the loss of PAI-1, and were highly upregulated in response to the addition of platelets. Genes in this cluster included those involved in tissue development, anatomical structure morphogenesis, cell proliferation, epithelial development, angiogenesis, and the positive regulation of cell migration. Cluster vi encompassed 222 genes that were highly downregulated in response to the loss of PAI-1. This cluster was composed of genes of the pathways concerning the response to viruses, type 1 interferon signaling, immune response, and the negative regulation of viral genome replication.

Analysis of siRNA compared to siNEG yielded significant down- and upregulation of 424 genes and 257 genes, respectively (Figure 2B). Pathways involving cell motility, locomotion, and regulation of cell migration were significantly downregulated when PAI-1 was transiently silenced, while glycosylation and glycoprotein biosynthesis were upregulated. Enrichment pathway analysis (Figure 2C) yielded significant downregulation of 13 molecular pathways. These included the regulation of cellular migration, phosphorylation, anatomical structure morphogenesis, intracellular signal transduction, cellular protein modification, and the stress-activated protein kinase signaling cascade. Significantly upregulated molecular pathways included glycosylation, protein glycosylation, homophilic cell adhesion *via* plasma membrane adhesion molecules, cell-cell adhesion, and the biosynthetic processes of several molecules including glycoproteins and carbohydrate-derived products. Functional categories that were significantly affected when PAI-1 was silenced included the Acetyl-CoA biosynthetic process, detection of virus, and regulation of cell migration. Functional categories from a related analysis included cell migration and cell motility, but also included categories such as regulation of response to stress, inflammatory response, and regulation of protein transport. Cross-referencing of significantly downregulated pathway lists revealed multiple functional categories returned by all analyses. Binary alignment map (BAM) files were uploaded to IGV and demonstrated alterations in gene expression in exons in cells where PAI-1 had been lost and where platelets had been added, and also in intronic regions when PAI-1 had been silenced (Additional File 5). A list of genes associated with cell migration was used to create a gene map (Figure 2D), which contained both known and predicted protein-protein interactions demonstrating that SERPIN E1 is central to many of the biological processes involved in ovarian cancer metastasis, including negative regulation of cell adhesion mediated by integrin, regulation of wound healing, positive regulation of interleukin-8 (IL-8) production, regulation of cell-matrix adhesion, regulation of cell migration, and positive regulation of angiogenesis. Our analysis of significantly upregulated genes yielded a list of 20 significantly enriched functional categories, which included protein glycosylation, cell-cell adhesion, and regulation of metallopeptidase activity.

**FIGURE 3:**
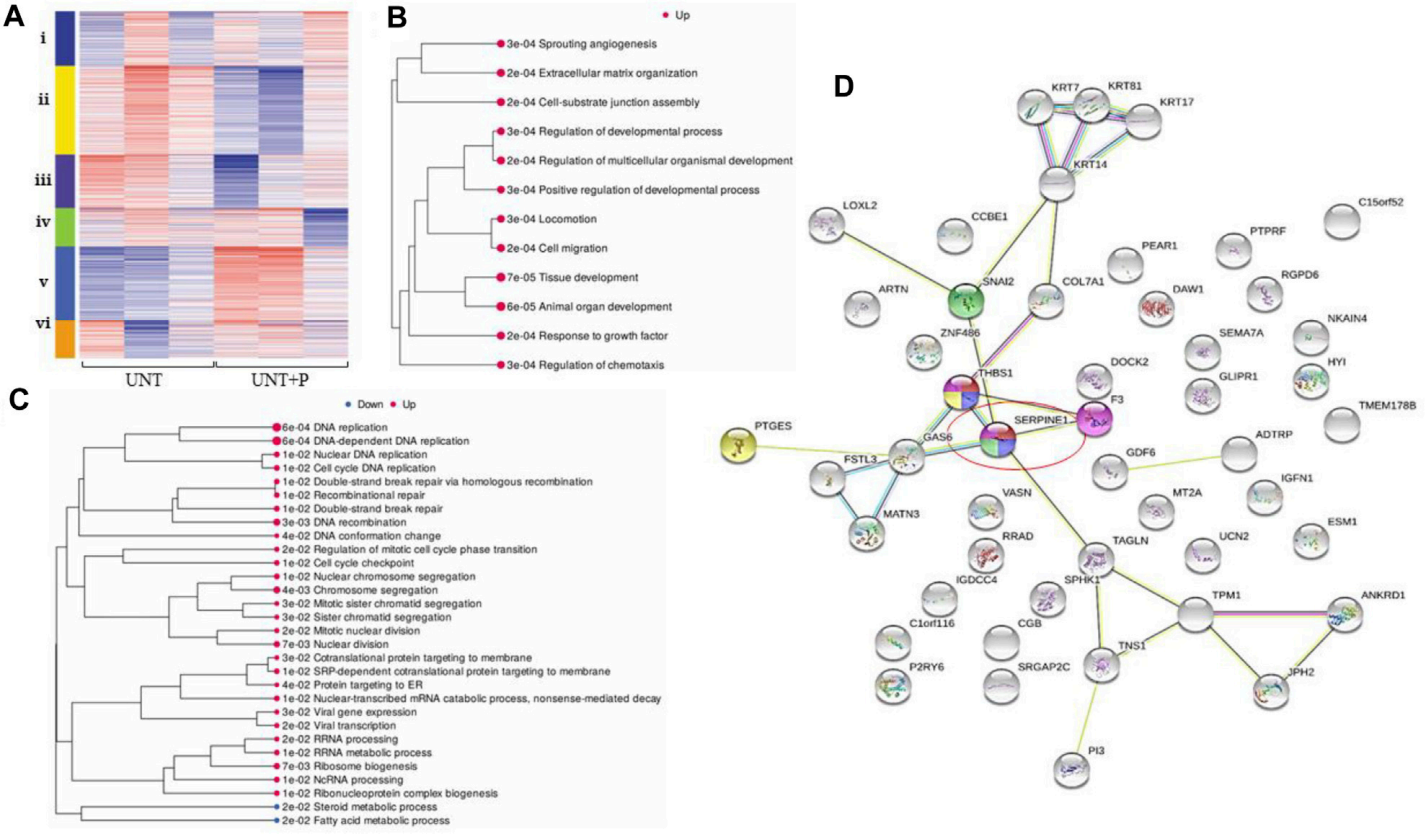
Platelet-cancer cell interactions, interrogated by RNA-Seq, increase gene expression along pathways identified as playing key roles in OC metastasis. For all heatmaps, up- and downregulation and are represented by red and blue, respectively, and for all treatment types, n = 3. Sequences were trimmed and corrected for potential batch effect using the ComBat-Seq negative binomial method. Analyses were performed using online resources, including Integrative Genomics Viewer (IGV, The Broad Institute, Cambridge, MA United States), iDEP92, ShinyGO and GSEA (Bioinformatics, South Dakota State University, SD, United States), and Search Tool for the Retrieval of Interacting proteins (STRING Consortium). *p*-values were adjusted after Benjamini–Hochberg correction. Significance criteria were an adjusted *p*-value less than or equal to 0.05, a minimum expression-fold change of 1.5, and a false discovery rate (FDR) of 0.05 or less. **(A).** Cluster analysis in iDEP92 shows significant pathway enrichment including: **i)** DNA replication, interstrand crosslink repair, **ii)** circulation, ion transport, **iii)** sterol biosynthesis, cholesterol metabolism, **iv)** cytokine signaling, neutrophil chemotaxis, **v)** cell migration, cell differentiation, and **vi)** inflammatory response, response to virus; **(B).** Significantly enriched pathways when platelets are added to OC cells comparing untreated cells to cell treated with platelets within the context of all treatment types with larger dots representing smaller *p*-values; **(C)**. Significantly enriched pathways when platelets are added to OC cells directly comparing untreated cells to cell treated with platelets with larger dots representing smaller *p*-values; **(D)**. Gene map generated by STRING illustrates that platelets influence HGSOC Metastasis *via* SERPIN E1 (circled).

SERPIN E1 was significantly upregulated when platelets were co-incubated with OC cells, with an expression-fold change of 2.04, and *p* ≤ 0.0001. A cluster analysis (Figure 3A) demonstrated significant dysregulation of the pathways integral to cell differentiation, extracellular matrix organisation, circulatory system process, response to virus, immune response, and leukocyte migration. Enrichment pathway analyses (Figs. 3B, 3C) demonstrated significant upregulation of many molecular pathways, including cell migration, regulation of cell migration, and animal organ development. Cross-referencing and subsequent analytics yielded 17 significantly upregulated pathways that included cell cycle DNA replication, negative regulation of interferon-gamma production, and interstrand crosslink repair, and also returned a list of 13 significantly downregulated pathways that included cholesterol metabolic process, response to type I interferon and type I interferon signaling. 30 functional categories significantly upregulated by the presence of platelets on ovarian cancer cells included cell migration, angiogenesis, regulation of response to wounding, and extracellular matrix organisation. A gene map of protein-protein interactions illustrated that SERPIN E1 is central to biological processes associated with these pathways and central to OC metastasis (Figure 3D).

An analysis of genes that were differentially expressed when platelets were added to SK-OV-3 cells where PAI-1 had been silenced returned 11 significantly upregulated genes and 6 significantly downregulated genes with no significant gene enrichment (not shown).

**FIGURE 4:**
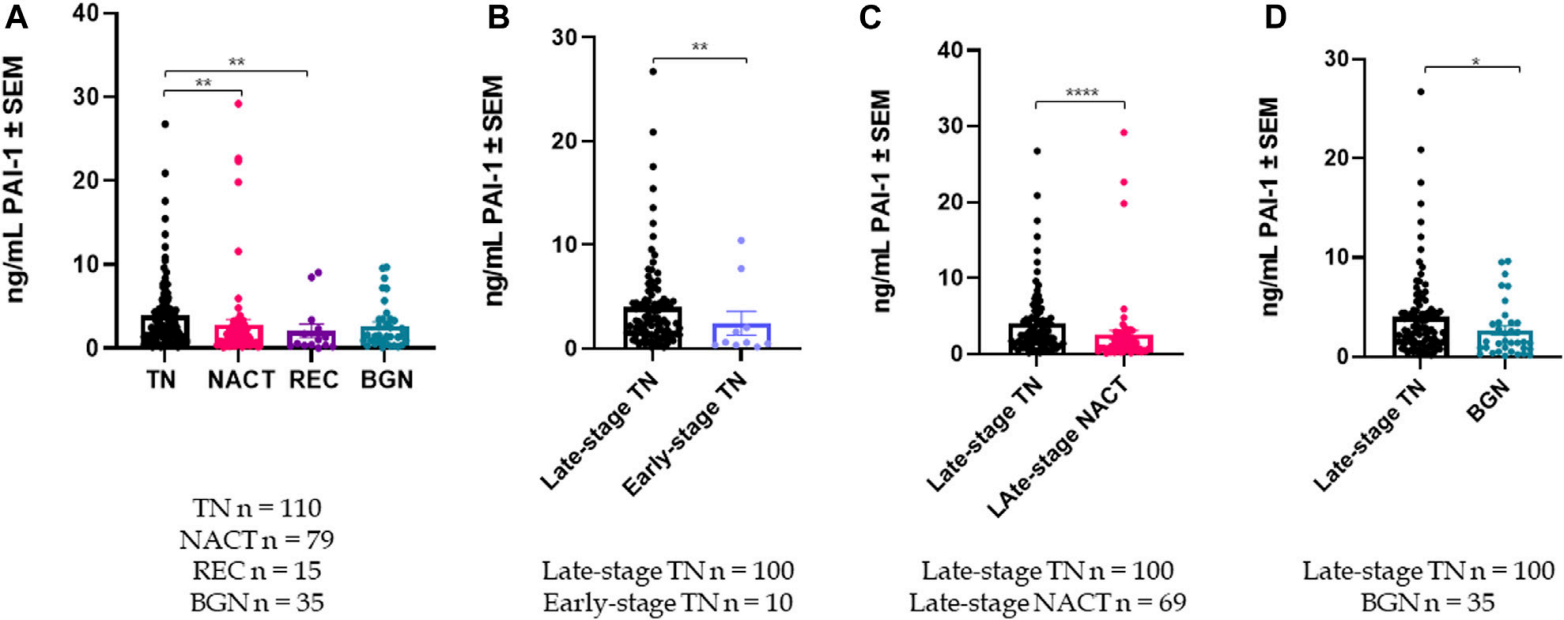
Plasma PAI-1 levels are significantly different among cohorts: **(A)** A one-way ANOVA of plasma PAI-1 across patient cohorts was significant with *p* = 0.0003, with *post hoc* significance between the TN (n = 110) and NACT (n = 79) cohorts, and also between the TN (n = 110) and Recurrent (n = 15) cohorts; **(B).** Plasma PAI-1 is significantly higher in TN patients with late-stage disease (n = 100) than in TN patients with early-stage disease (n = 10) (2-tailed *t*-test, *p* = 0.0081); **(C)**. Plasma PAI-1 is significantly reduced by neoadjuvant chemotherapy (*t*-test, *p* ≤ 0.0001); **(D).** Plasma PAI-1 is significantly higher in TN patients with late-stage disease (n = 100) than in patients with benign serous histology (n = 35) (2-tailed *t*-test, *p* = 0.0112). *p ≤ 0.05, **p ≤ 0.01, ****p ≤ 0.0001.

**FIGURE 5:**
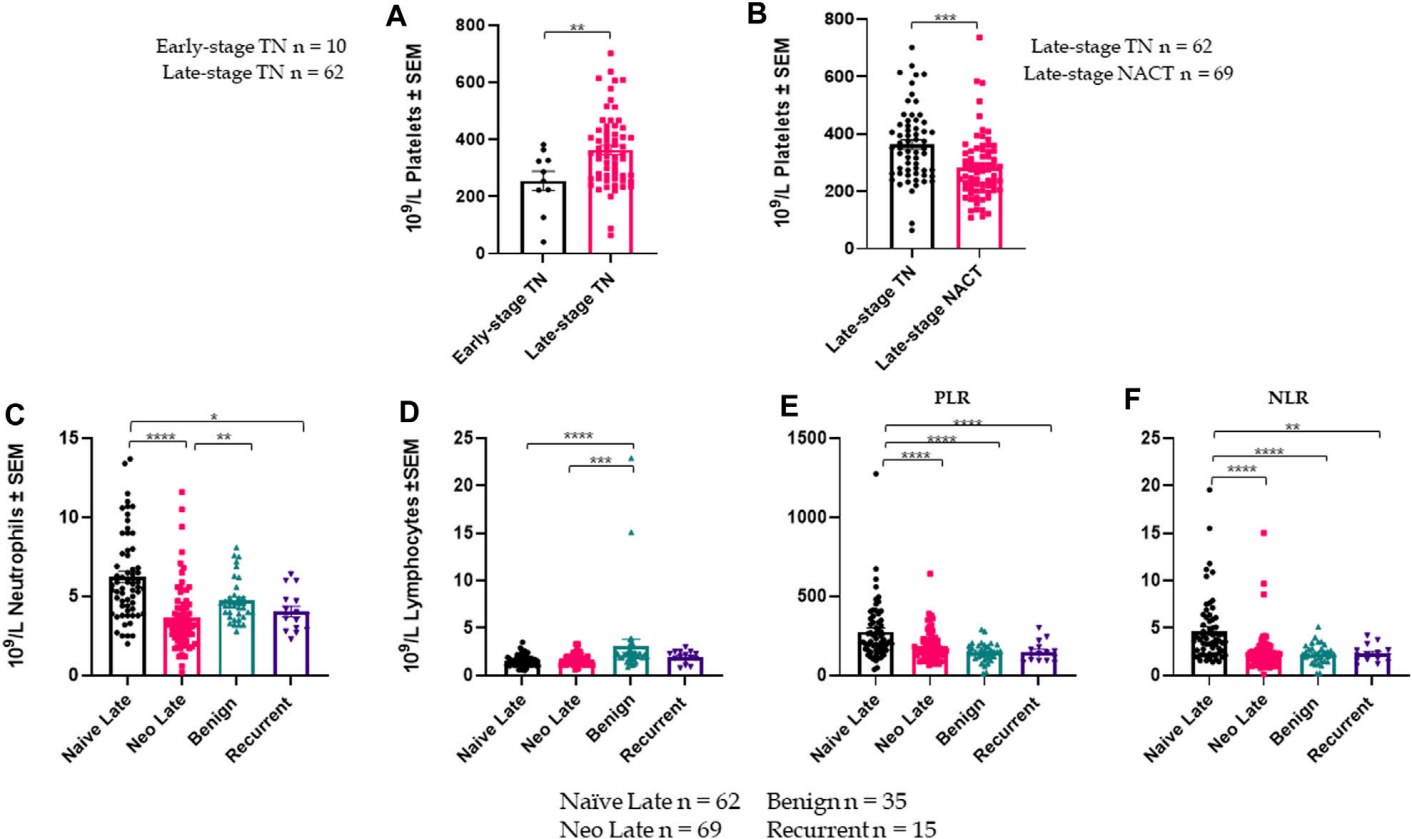
Platelet, lymphocyte, and neutrophil data across cohorts are shown +/- standard error of the mean (SEM). n for each cohort represents available data and differ from numbers in Fig.4: **(A)**. Mean platelets are significantly higher in TN patients with late-stage disease (n = 62) than in TN patients with early-stage disease (n = 10) (2-tailed *t*-test, *p* = 0.0068); **(B)**. Mean platelets are significantly lower in NACT patients with late-stage disease (n = 69) than in TN patients with late-stage disease (n = 62) (2-tailed *t*-test, *p* = 0.0004); **(C)**. A one-way ANOVA of mean neutrophils across patient cohorts (including late-stage only for TN and NACT) was significant with *p* ≤ 0.0001; **(D)**. A one-way ANOVA of mean lymphocytes across patient cohorts (late-stage only) was significant with *p* = 0.0089; **(E)**. A one-way ANOVA of platelet-to-lymphocyte ratios (PLR) across patient cohorts (late-stage only for TN and NACT) was significant with *p* ≤ 0.0001; **(F)**. A one-way ANOVA of neutrophil-to-lymphocyte ratios (NLR) across patient cohorts (late-stage only for TN and NACT) was significant with *p* ≤ 0.0001. *p ≤ 0.05, **p ≤ 0.01, ***p ≤ 0.001, ****p ≤ 0.0001.

***Plasma PAI-1 is a Marker of Treatment Response in Patient Cohorts*** Cohort demographics are reported in Table 4, with reference ranges for hematology listed in Additional File 6. Plasma PAI-1 values for TN (n = 110), NACT (n = 69), recurrent (n = 15), and benign (n = 35) cohorts are reported in Table 5. There is a difference in patient numbers (n) between Table 4 and Table 5 because Table 5 includes only pathological late-stage NACT patients that were used for statistical analyses. A one-way ANOVA across cohorts where TN and NACT cohorts were inclusive of both early- and late-stage patients was significant (Figure 4A, *p* = 0.0003), with Tukey’s *post hoc* significance between the TN and NACT cohorts (*p* = 0.0034) and the TN and recurrent cohorts (*p* = 0.0038). Two-tailed t-tests revealed that plasma PAI-1 was significantly higher in TN patients with late-stage disease (4.0 ng/mL) than in TN patients with early-stage disease (2.4 ng/mL) (Figure 4B, *p* = 0.0081), significantly lower in patients with late-stage disease in the NACT cohort (2.50 ng/mL) than in patients with late-stage disease in the TN cohort (Figure 4C, *p* ≤ 0.0001), and was significantly lower in patients with benign serous histology (2.6 ng/mL) than in TN patients with late-stage disease (Figure 4D, *p* = 0.0112). Patient numbers (n) differ between PAI-1 analyses in Figure 4 and platelet, neutrophil, and lymphocyte analyses in Figure 5 due to the availability of blood data for some but not all members of our treatment-naïve cohort. Mean platelet counts for patients in all cohorts are reported in Table 5. A positive linear relationship was noted between platelets and stage in the TN cohort (not shown). A two-tailed *t*-test yielded significantly lower mean platelets in TN patients with early-stage disease than in those with late-stage disease (Figure 5A, *p* = 0.0068), and second two-tailed *t*-test also showed significantly lower mean platelets NACT patients with pathological late-stage disease than in TN patients with late-stage disease (Figure 5B, *p* = 0.0004). 19% of TN patients with late-stage disease exhibited thrombocytosis (≥450 × 10^9^ platelets/L) *versus* none of the TN patients with early-stage disease, and only 7% of patients with pathological late-stage disease in the NACT cohort. Mean neutrophils and lymphocytes for each cohort are listed in Table 5, as well as platelet-to lymphocyte (PLR) and neutrophil-to-lymphocyte (NLR) ratios. Mean neutrophils were significantly higher in TN patients with late-stage disease than in both NACT patients with pathological late-stage disease (Figure 5C, *p* ≤ 0.0001) and patients with recurrent disease (Figure 5C, *p* = 0.0222). Mean neutrophils in NACT patients with pathological late-stage disease were significantly lower than in the benign cohort (Figure 5C, *p* = 0.0039). Mean lymphocytes were significantly lower in TN patients with late-stage disease than in the benign cohort (Figure 5D, *p* ≤ 0.0001) and were also significantly lower in NACT patients with pathological late-stage disease than in the benign cohort (Figure 5D, *p* = 0.0003). Mean PLR was significantly higher for patients with late-stage disease *versus* those with early-stage disease in the TN cohort (Figure 5E, *p* = 0.0067). Mean PLR was significantly higher for patients with late-stage disease in the TN naïve cohort than for patients with pathological late-stage disease in the NACT cohort (Figure 5E, *p* < 0.0001), patients in the benign cohort (Figure 5E, *p* < 0.0001), and patients with recurrent disease (Figure 5E, *p* < 0.0001). Mean NLR was significantly higher in the TN cohort than in the NACT cohort (Figure 5F, *p* ≤ 0.0001), and also significantly higher than in both benign (Figure 5F, *p* ≤ 0.0001) and recurrent cohorts (Figure 5F, *p* = 0.0047). Platelets did not correlate with plasma PAI-1 in the TN cohort (Additional File 7), and a positive Pearson correlation was observed between plasma PAI-1 and neutrophil counts in the TN cohort (Additional File 7). Preliminary CTC data are reported in Additional File 8.

**TABLE 4:** Patient demographics for treatment-naïve (TN, neoadjuvant chemotherapy (NACT), recurrent and benign cohorts.

Variable	n	TN	NACT	Recurrent	Benign
Total Patients (%)	239	110 (46.0%)	79 (33.0%)	15 (6.3%)	35 (14.7%)
Mean Age (*SD*)	239	63.8 (*11.4*)	62.9 (*11.3*)	57.9 (*12.8*)	59.6 (*13.1*)
Menopausal Status	124				
Postmenopausal (%)	104 (83.9%)	33 (86.8%)	43 (95.6%)	5 (71.4%)	23 (67.6%)
Premenopausal (%)	20 (16.1%)	5 (13.2%)	2 (4.4%)	2 (28.6%)	11 (32.4%)
Stage					
1	11	4	7	n/a	n/a
2	9	6	3	n/a	n/a
3	126	63	63	n/a	n/a
4	43	37	6	n/a	n/a
					
Mean BMI (*SD*)	128	27.4 (*6.0*)	26.6 (*5.0*)	30.6 (*6.5*)	29.3 (*7.4*)

**TABLE 5:** Platelets, neutrophils, lymphocytes, platelet-to-lymphocyte ratio, neutrophil-to-lymphocyte ratio, and plasma PAI-1 for early- and late-stage TN, late-stage NACT, recurrent, and benign cohorts are shown as mean (*SD*) values. “y” indicates surgical staging post NACT. Significance is indicated for differences between cohorts. *p ≤ 0.05, **p ≤ 0.01, ***p ≤ 0.001, ****p ≤ 0.0001.

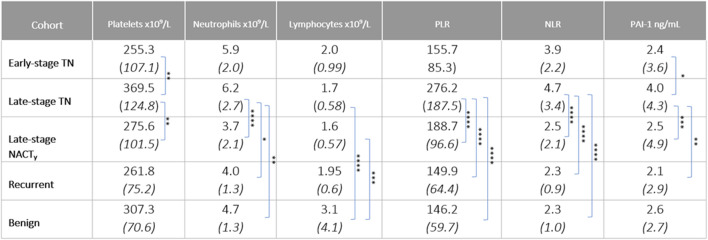

## Discussion

*In vitro* assay results support current evidence that platelets drive PAI-1 production in platelet-cloaked ovarian cancer cells (Spillane et al., 2021). This is relevant in the context of CTCs, where platelets protect against shear stress (Egan et al., 2014), contribute to immune evasion (Nieswandt et al., 1999), and assist in the hematogenous metastasis of OC (Labelle et al., 2014; Pradeep et al., 2014). Platelets were unable to restore PAI-1 post silencing, demonstrating that although platelet-cancer cell interactions trigger PAI-1 transcription, increased PAI-1 is not merely an additive effect *via* platelet degranulation, but involves a molecular mechanism by which the platelets and cancer cells interact which results in the transcription and translation of intracellular PAI-1 in the cancer cells that may be excreted into the tumor microenvironment and the circulation. The role of TGF-β in PAI-1 transcription was previously illustrated by Dennler *et al* (Dennler et al., 1998); however, just as Kirschbaum *et al* demonstrated liver regeneration *via* horizontal transfer of platelet mRNA to hepatocytes (Kirschbaum et al., 2015), horizontal transfer of PAI-1 mRNA to cancer cells from the platelets cannot be ruled out, and PAI-1 may serve as a transcription factor (TF) for itself in this scenario. The exact mechanism by which this occurs has yet to be elucidated and requires further research. The addition of platelets and their releasate to SK-OV-3 cells resulted in an increase in cellular proliferation, as demonstrated *via* flow cytometry. This parallels previous research by Giacoia supporting the mitogenic potential of PAI-1 (Giacoia et al., 2014), in which it was demonstrated that overexpression of PAI-1 increased cell proliferation by “pushing” cells into S phase, effectively bypassing DNA mutation and repair checkpoints. Conversely, Giacoia found that silencing PAI-1 inhibited cellular proliferation and led to an increase in the number of cells in the G_0_/G_1_ phase, and a corresponding decrease in the number of cells in S phase. The proliferation observed in our cell-cycle analysis in the presence of platelets and their releasate aligns with this research, linking PAI-1 to cancer cell proliferation, however further research is required. Our results demonstrated an inverse relationship between wound-edge migration and proliferation, and in conjunction with significantly impaired migration and invasion when PAI-1 is lost, this suggests that increased migration and proliferation may result from an increase in PAI-1. Increased invasion was observed when platelets were added to SK-OV-3 cells, however results were not significant as these cells are highly invasive at baseline. Taken together, these results provide a foundation for a more finely-tuned investigation into the molecular mechanisms of platelet-cancer cell interactions in the context of PAI-1. SERPIN E1, the gene that codes for PAI-1, is central to many molecular pathways that underpin OC metastasis, and this was demonstrated by significant downregulation of those pathways when PAI-1 had been silenced. Notable among these was Acetyl-CoA production, suggesting that PAI-1 plays a role in cancer cell metabolism. Importantly, the phenotypic changes associated with metastasis were mirrored by our transcriptomic results–the genotype matched the phenotype. Significant downregulation of mechanisms associated with phosphorylation was observed when PAI-1 was silenced, and has potential implications for protein function and signal transduction, inflammation, apoptosis, molecule transport across cell membranes, and - crucially - oxidative phosphorylation, which when coupled with the dysregulation of Acetyl-CoA production further implicates PAI-1 in cancer cell metabolism. Silencing PAI-1 further resulted in the significant upregulation of several distinct pathways, including glycosylation *via* increased expression of glucoside xylosyltransferase (GXYLT), which when silenced has been shown to increase Notch expression and activity (Lee et al., 2013). This suggests that silencing PAI-1 could decrease Notch activity, thereby suppressing angiogenesis. Additionally, significant upregulation of Wnt4 in our knockdown suggests that PAI-1 may be a regulator of non-canonical signaling in OC (Bernard et al., 2008; Teeuwssen and Fodde, 2019). Significant downregulation of homologous recombination when PAI-1 was silenced evidences a role for PAI-1 in decoding chemoresistance. Connections between platinum and DNA determine how particular segments of DNA are presented for repair or are occluded (de Sousa et al., 2014), and the platinum adducts themselves may be responsible for the loss of the very DNA repair mechanisms upon which they rely. Excision repair cross-complementation group 1 (ERCC1) protein in the nucleotide excision repair (NER) pathway is thought to be an important protein in the use of platinum-based chemotherapeutic agents (Mittica et al., 2018). This could affect treatment options with Poly-ADP-ribose polymerase (PARP) inhibitors, which affect base-excision repair (BER) pathways in patients both with and without breast cancer gene (BRCA) mutations by forcing non-homologous end joining (NHEJ) (Teliga-Czajkowska et al., 2019). Our transcriptomic analysis also revealed that co-incubation of platelets with OC cells significantly increased the number of SERPIN E1 transcripts, mirroring results seen *in vitro* in our RT-qPCR results. Upregulation of interstrand crosslink repair in OC cells in the presence of platelets demonstrates a potential “see-saw” effect corresponding to the significant downregulation of homologous recombination observed when PAI-1 was silenced. This could provide insight into the genesis of chemoresistance and warrants further research, particularly as mutations that emerge and persist when cell-cycle checkpoints are dysfunctional have been observed in conjunction with the upregulation of PAI-1 (Giacoia et al., 2014). Furthermore, platelet-induced PAI-1 activity may function on a gradient, and paired with significant upregulation of anatomical structure morphogenesis when OC cells were co-incubated with platelets, it does not seem unreasonable to view PAI-1 not only as a mitogenic factor, but also as a potential morphogen. After establishing proof of concept with our metastatic OC cell line model, we moved our study directly into several patient cohorts to investigate the translational potential of PAI-1 as a biomarker. Indeed, our results align with previous research which links increased plasma PAI-1 to advanced ovarian disease (Pappot et al., 1995); however, our focus on the high-grade serous histological subtype is both novel and critical. HGSOC is the most lethal gynecological malignancy, yet to date, research has been conducted in tumor and stromal tissue without stratified histological analyses. It is crucial to consider HGSOC as a separate entity. Chambers *et al* found that 50% of both primary and metastatic tumors expressed PAI-1, that patients with late-stage disease had better overall survival when their primary tumors were negative for PAI-1, and that high PAI-1 plays a protective role for OC (Ho et al., 1999). Others have linked PAI-1 to poor prognosis in OC patients (Peng et al., 2019; Teliga-Czajkowska et al., 2019), and have also shown that elevated baseline plasma PAI-1 is a predictor of relapse in breast cancer (BC) patients (Ferroni et al., 2014). Discrepancies in the behavior of PAI-1 in angiogenesis and apoptosis serve to highlight its dual nature (Balsara and Ploplis, 2008; Placencio and DeClerck, 2015) and provide evidence that its functionality may indeed be concentration-dependent. 4G/5G polymorphism has been associated with colorectal and endometrial cancers (Wang et al., 2013), although implications of these allelotypes are unclear relative to OC. Two existing studies have not shown the homozygous 4G allelotype to be a risk factor in OC (Türkmen et al., 1997; Bentov et al., 2009), but investigation into genetic factors which may directly impact PAI-1 expression, including promoter polymorphisms, may be valuable with regard to patient treatment outcomes. As observed in our *in vitro* assays, when PAI-1 is lost, platelets alone do not restore it, and this further supports that baseline PAI-1 levels may play a role in the amount of PAI-1 that is produced as a result of the platelet-cancer cell interactome. It is noteworthy that mean plasma PAI-1 in our benign cohort was similar to that of patients in our NACT cohort (2.6 ng/mL and 2.8 ng/mL, respectively). Although there was a larger standard deviation in PAI-1 values in the NACT cohort than in the benign cohort (5.2 vs 2.7), this does suggest that patients undergoing NACT may have had their PAI-1 levels reduced to pre-malignancy levels and provides justification for further research. Studies by others have demonstrated that PAI-1 in fluid from malignant, borderline, and benign tumors is highest in malignant tumors (Boss et al., 2002). In a study by Casslén, plasma PAI-1 showed no significant difference between benign and malignant tumors in a pre-menopausal cohort (Casslén et al., 1994), although this was higher than in healthy controls. A post-menopausal cohort in that same study demonstrated that the presence of malignant tumors with ascites in patients resulted in significantly higher plasma PAI-1 than in patients who had malignant tumors without ascites, patients with benign histology, or healthy controls (Casslén et al., 1994). These results were not stratified by histological subtype, and are therefore difficult to interpret and align with our data, however they do provide evidence that PAI-1 is significantly associated with advanced disease. Platelet-cancer cell interactions upregulate PAI-1 in a metastatic OC cell line model - the result of a platelet-cancer cell interactome–and contribute to a metastatic phenotype underpinned and mirrored by a metastatic genotype. PAI-1 did not correlate with patient platelet counts in our TN cohort, and this distinct absence of correlation is crucial as it supports the hypothesis that plasma PAI-1 levels occur independently of platelet PAI-1. This also supports previous research which demonstrated that there was no correlation between platelet count in platelet-rich plasma (PRP) and PAI-1 antigen from platelet-free plasma (PFP) (Booth et al., 1988). Neutrophils are recruited into tissue by IL-8 (Romani de Wit et al., 2003). PAI-1 is required to stabilize IL-8 during transendothelial migration of circulating neutrophils to sites of inflammation, and platelet PAI-1 is not a factor in this (Marshall et al., 2003), suggesting that the inflammasome created by intravasating ovarian tumor cells may also contribute to increased plasma PAI-1. The significant correlation between plasma PAI-1 and neutrophils in our TN cohort supports PAI-1’s involvement in both inflammatory and immune responses. Platelet cloaking of cancer cells potentiates a mesenchymal phenotype (Guo et al., 2019) in which PAI-1 may serve as a TF for itself (Kirschbaum et al., 2015). Cancer cells undergoing EMT experience an increase in PAI-1 expression (Nakatsuka et al., 2017), and this can aid in pre-metastatic events with platelets further assisting in the establishment of micrometastases (Labelle et al., 2014; Pradeep et al., 2014). PAI-1 expression is induced during EMT, tissue repair, and cancer cell dissemination (Kalluri and Weinberg, 2009), and it is not out of the question to suggest that PAI-1 functions as a morphogen within the scope of cancer genesis, progression, and metastasis, including in HGSOC, and that platelets contribute to the gradient on which PAI-1 operates, tempered by neutrophil recruitment within the inflammasome. PAI-1 may be targetable by small-molecule inhibitors, as seen with IMD-4482 (Nakatsuka et al., 2017) and TM5275 (Mashiko et al., 2015), however, treatment may hinge upon individual PAI-1 allelotype and disease stage, which also allude to a functional gradient. It has been observed that PAI-1 directly inhibits mitochondrial function, facilitating the switch from oxidative phosphorylation to glycolytic metabolism in TNBC (Humphries et al., 2020), and therefore continued investigation into PAI-1, its potential position in excision repair pathways, including NER and BER, along with its involvement in Acetyl-CoA production, is integral to understanding cancer metabolism and chemoresistance. OC is severely understudied and no screening exists, therefore it is crucial that we explore all possible avenues that could give us clues to the molecular behaviors of that which often leads to poor outcome. The research contained herein adds to a growing body of evidence demonstrating PAI-1’s involvement in OC metastasis, and our results provide a foundation upon which can be built an understanding of the molecular mechanisms underlying HGSOC in the context of metastasis, treatment, and of progressive disease, but more research is required in order to delineate these mechanisms more clearly. SK-OV-3 was the sole cell line used in our *in vitro* assays, and while this was a limitation of this study, the main focus of this project was to demonstrate the translational relevance of elevated PAI-1 and the interactions between platelets and cancer-cells in HGSOC patient cohorts, which we have accomplished. As a highly metastatic epithelial ovarian adenocarcinoma expressing PAI-1 (Nakatsuka et al., 2017), SK-OV-3 cells were deemed the most appropriate cell line for our *in vitro* research. Other available in-house serous cell lines such as OAW-42 and OVCAR3 were not deemed appropriate for this study as no PAI-1 was detected by RT-qPCR. Further interrogation of our CTC cohort is warranted in order to more accurately characterize HGSOC, and *ex vivo* culturing and establishment of CTC cell lines, while complex, would further advance the evidence we provide here. Studies by our group regarding the isolation and culturing of CTCs are ongoing as part of the All Ireland Liquid Biopsy Consortium (CLuB). *In vivo* testing of circulating ovarian tumor cells in the contexts of both thrombocytosis and thrombocytopenia could assist in more accurately pinpointing PAI-1’s position in the metastatic cascade. These studies would allow for a more in-depth investigation of molecular interactions and platelet-cancer cell interactions involving overexpression of PAI-1, including overall effects locally with regards to the initial tumor site, behaviors within the circulatory environment, and the development of secondary metastases, whether proximal or distant; however, this was beyond the scope of this paper. Consideration of fallopian tube origin would also need to be undertaken when choosing an appropriate *in vivo* model. Pradeep *et al* demonstrated hematogenous metastasis of OC to the omentum as a result of an ErbB3/NRG1 signaling axis (Pradeep et al., 2014), and in the results of our RNA-Seq analysis, it was observed that NRG1 was significantly downregulated when PAI-1 was silenced. Indeed, interrogation of any potential effect of PAI-1 on this axis could yield more clues in mapping the metastatic cascade. Our work here provides evidence of a central role for PAI-1 in the platelet-cancer cell interactome, and demonstrates its potential as a biomarker, gauge of therapy response, prognostic indicator, therapeutic target, and guide to deciphering the molecular complexities of OC.

## Figures and Tables

**Table:** 

***a* bar**	Average Acceleration
**aa**	Amino Acids
**ACD**	Acid Citrate Dextrose
**ANOVA**	Analysis of Variance
**BAM**	Binary Alignment Map
**BC**	Breast Cancer
**BCA**	Bicinchoninic Acid
**BER**	Base-excision Repair
**BRCA**	Breast Cancer Gene
**CaCl2**	Calcium Chloride
**cDNA**	Copy Deoxyribonucleic Acid
**CTCs**	Circulating Tumor Cells
**CWIUH**	Coombe Women and Infants University Hospital
**DEGs**	Differentially-expressed Genes
**DPBS**	Dulbecco’s Phosphate-buffered Saline
**ECM**	Extracellular Matrix
**EDTA**	Ethylenediaminetetraacetic acid
**EHS**	Engelbreth-Holm-Swarm
**ELISA**	Enzyme-linked Immunosorbent Assay
**EMT EpCAM**	Epithelial-to-mesenchymal Transition Epithelial Cellular Adhesion Molecule
**EOC**	Epithelial Ovarian Cancer
**ERCC1**	Excision Repair Cross-complementation Group 1
**FBS**	Fetal Bovine Serum
**FDR**	False Discovery Rate
**FM**	Fresh Medium
**GXYLT**	Glucoside Xylosyltransferase
**h**	Hours
**HGSOC**	High-grade Serous Ovarian Cancer
**IGV**	Integrative Genomics Viewer
**IL-8**	Interleukin 8
**kDa**	KiloDalton
**LCS**	Lipofectamine Complex Solution
**LPR1**	Lipoprotein Receptor-related Protein 1
**min**	Minutes
**mm**	Millimeters
**mm** ^**2**^	Millimeters Squared
**mRNA**	Messenger RNA
**NACT**	Neoadjuvant Chemotherapy
**NER**	Nucleotide Excision Repair
**NHEJ**	Non-homologous End Joining
**NLR**	Neutrophil-to-lymphocyte Ratio
**OC**	Ovarian Cancer
**P/PLT**	Platelets
**PAI-1**	Plasminogen Activator Inhibitor 1
**PARP**	Poly-ADP-ribose Polymerase
**PFP**	Platelet-free Plasma
**PGE2**	Prostaglandin 2
**PLR**	Platelet-to-lymphocyte Ratio
**PRP**	Platelet-rich Plasma
**PTE**	Polyethylene terephthalate
**RCF**	Relative Centrifugal Force
**REL**	Platelets/Releasate
**RPM**	revolutions per minute
**RT-qPCR**	Real-time Quantitative Polymerase Chain Reaction
**sec**	Seconds
**SERPIN E1**	Serine Protease Inhibitor 1
**siNEG**	Negative Control siRNA
**siRNA**	Small Interfering Ribonucleic Acid
**SMB**	Somatomedin
**STRING**	Search Tool for the Retrieval of Interacting Proteins
**TF**	Transcription Factor
**TGF-β**	Transforming Growth Factor β
**TN**	Treatment-naïve
**tPA**	Tissue-type Plasminogen Activator
**UNT**	Untreated
**uPA**	Urokinase-type Plasminogen Activator
**uPAR**	Urokinase-type Plasminogen Activator Receptor
***v* bar**	Average Velocity
**VTE**	Venous Thrombotic Event
**Δt**	Change in Time
**Δv**	Change in Velocity
**μm/h**	Micrometers per Hour
**μm/h/h**	Micrometers per Hour per Hour
**μm/h** ^**2**^	Micrometers per Hour Squared
**σ**	Standard deviation

## Data Availability

The datasets presented in this study can be found at: https://0-www-ncbi-nlm-nih-gov.brum.beds.ac.uk/geo/query/acc.cgi?acc=GSE223654.
