# Supplementary material for: Plasminogen activator inhibitor 1 is associated with high-grade serous ovarian cancer metastasis and is reduced in patients who have received neoadjuvant chemotherapy
Source: Front Cell Dev Biol. 2023 Dec 7;11:1150991. doi: 10.3389/fcell.2023.1150991 (PMC10740207; doi:10.3389/fcell.2023.1150991)
Supplement: Supplementary file 1 [file DataSheet7.PDF]

Additional File 7

Correlation Analyses

Analyses were run to see if any correlations existed between platelets and plasma PAI-1 or between neutrophils and PAI-1.

A

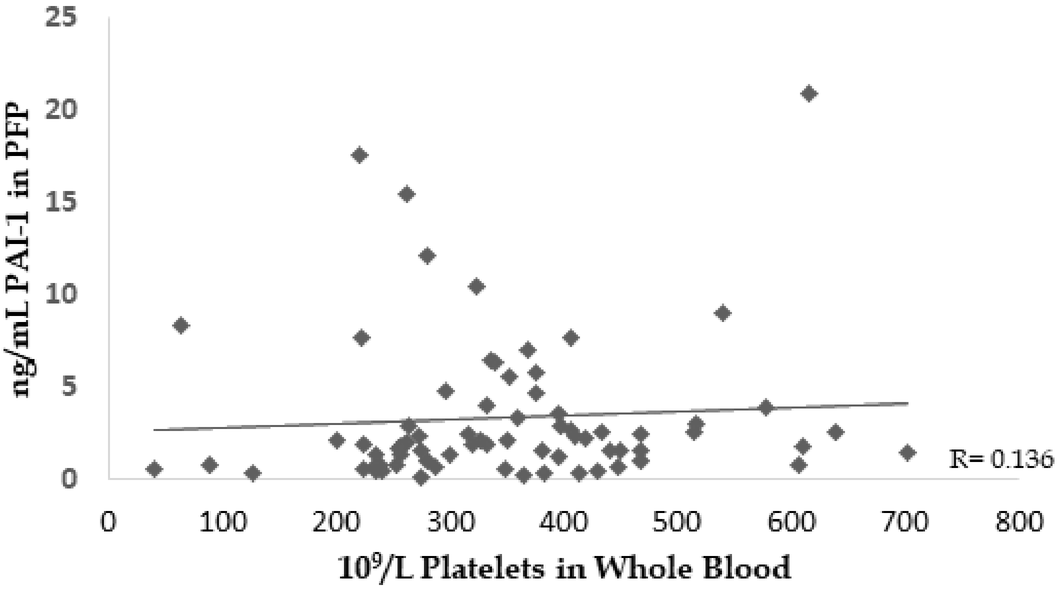

B

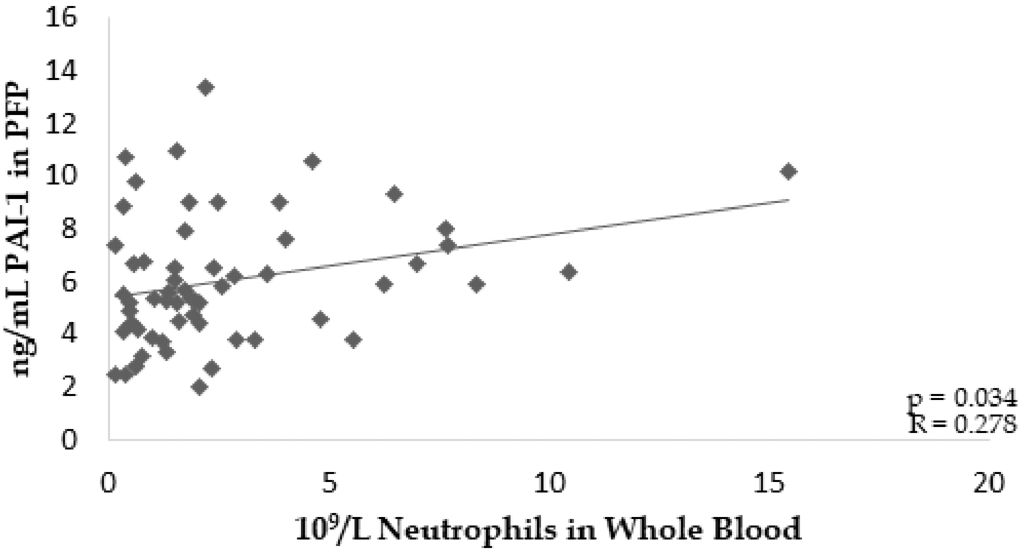

A Platelets do not correlate with plasma PAI-1; **B** Neutrophils positively correlate with plasma PAI-1.
