# Supplementary material for: Plasminogen activator inhibitor 1 is associated with high-grade serous ovarian cancer metastasis and is reduced in patients who have received neoadjuvant chemotherapy
Source: Front Cell Dev Biol. 2023 Dec 7;11:1150991. doi: 10.3389/fcell.2023.1150991 (PMC10740207; doi:10.3389/fcell.2023.1150991)
Supplement: Supplementary file 2 [file DataSheet4.PDF]

Additional File 4

Immunofluorescence (IF) Staining

SK-OV-3 cells were seeded at a concentration of 2 x 10^4 per chamber in supplemented medium in an 8-chamber slide (Merck), and the slide was incubated at 37°C to allow adhesion. Medium was then replaced with antibiotic-free medium, and cells treated with siNEG, PAI-1 siRNA or Opti-MEM, (Gibco, USA), with siRNA prepared as previously described. After 24 h, half of the chambers received platelets isolated as previously described at a ratio of 1000 platelets per cell. After incubation, the cells were fixed in 100% methanol (Acros Organics), blocked in bovine serum albumin (BSA; Sigma, USA) in PBST (PBS+ 0.1% Tween 20), and stained with the anti-PAI-1 monoclonal antibody (mAb) mAb-33H1F7 (Thermo MA1- 40224). All wells were subsequently stained with AlexaFluor 488 goat anti-mouse secondary antibody (Invitrogen IgG H+L AlexaFluor 488 A11001) and Höechst 33342 (Thermo Pierce). Fluorescence microscopy was performed using the Nikon Eclipse TE300.

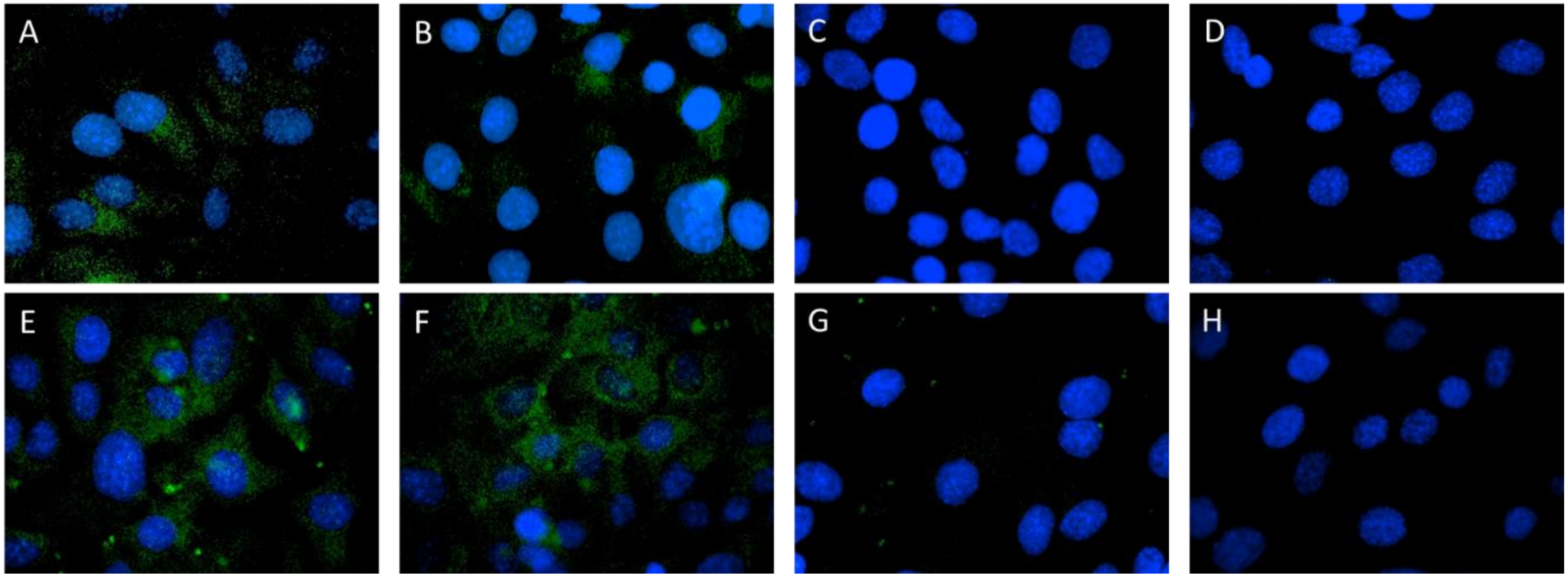

PAI-1 protein in SK-OV-3 cells visualized by fluorescence microscopy at 40x. All wells were incubated with a 1:500 dilution of mAb-33H1F7 (monoclonal murine IgG1 against PAI-1), 10µg/mL Höechst (seen in blue) and a 1:1000 dilution Alexa Fluor 488–conjugated goat anti-mouse IgG (seen in green), with the exception of wells D and H, which received Höechst and fluorophore-conjugated secondary IgG only. **A.** Untreated cells, **B**. Cells treated with negative control, **C.** Cells treated with PAI-1 siRNA, **D.** Untreated cells, **E.** Untreated cells incubated with platelets for 24h, **F.** Cells treated with siNEG (inert siRNA) and incubated with platelets for 24h, **G.** Cells treated with PAI-1 siRNA and incubated with platelets for 24h, **H.** Untreated cells incubated with platelets for 24h.
