# Supplementary material for: Plasminogen activator inhibitor 1 is associated with high-grade serous ovarian cancer metastasis and is reduced in patients who have received neoadjuvant chemotherapy
Source: Front Cell Dev Biol. 2023 Dec 7;11:1150991. doi: 10.3389/fcell.2023.1150991 (PMC10740207; doi:10.3389/fcell.2023.1150991)
Supplement: Supplementary file 3 [file DataSheet6.PDF]

Additional File 6

Table AF Hematology reference ranges

Factor	*Range*	*Unit*
Platelets	140-450	10^9/L
Neutrophils	2-7.5	10^9/L
Lymphocytes	1.5-3.5	10^9/L
NLR	< 2.8	
PLR	< 160	
