# Supplementary material for: Plasminogen activator inhibitor 1 is associated with high-grade serous ovarian cancer metastasis and is reduced in patients who have received neoadjuvant chemotherapy
Source: Front Cell Dev Biol. 2023 Dec 7;11:1150991. doi: 10.3389/fcell.2023.1150991 (PMC10740207; doi:10.3389/fcell.2023.1150991)
Supplement: Supplementary file 4 [file DataSheet3.PDF]

Additional File 3

Cell Cycle Analysis

Cells were seeded and treated in an identical manner to that described for the wound-healing assay, and at each time point 0 h, 6 h, 20 h, and 24 h cells were washed in DPBS, dissociated with trypsin-EDTA, and fixed by methanol according to the methods described by Pozarowski and Darzynkiewicz. Cells were then washed and resuspended in propidium iodide (PI) staining solution (supplemental). Flow cytometry was performed using Becton-Dickinson's FACS-Melody and analysed using FlowJo (Becton-Dickinson, USA).

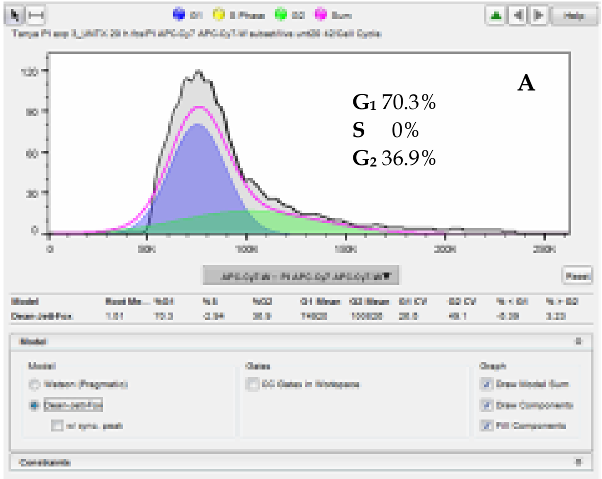

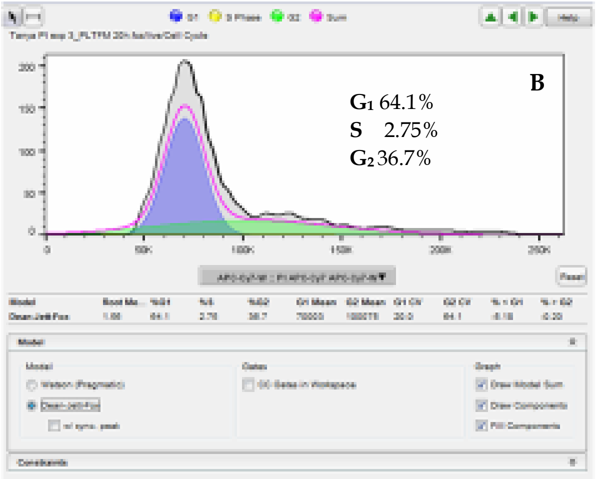

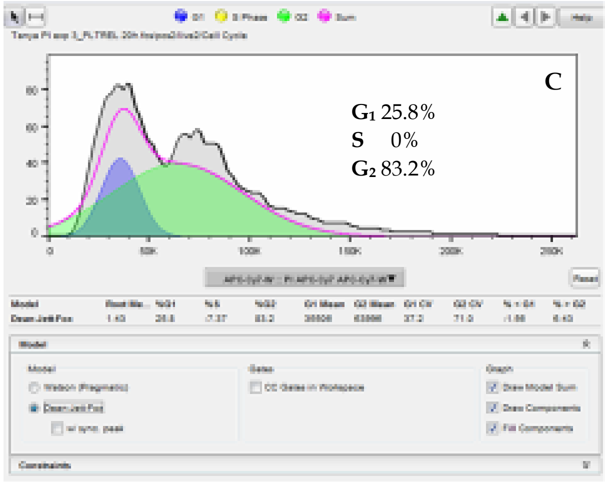

t = 20h, histograms of sample set 1 (of 3) demonstrate that a greater number of cells that were treated with platelets and then releasate are in G_2 phase than the other treatment types: **A.** UNT **B.** PLT/FM **C.** PLT/REL. G_1 is seen in blue, S is shown in yellow, and G_2 is shown in green.

Pozarowski, Piotr and Zbigniew Darzynkiewicz . "Analysis of cell cycle by flow cytometry " Methods in Molecular Biology. Vol. 281, pp 301-311. 2004.
