# Supplementary material for: Plasminogen activator inhibitor 1 is associated with high-grade serous ovarian cancer metastasis and is reduced in patients who have received neoadjuvant chemotherapy
Source: Front Cell Dev Biol. 2023 Dec 7;11:1150991. doi: 10.3389/fcell.2023.1150991 (PMC10740207; doi:10.3389/fcell.2023.1150991)
Supplement: Supplementary file 5 [file DataSheet1.PDF]

Additional File 1

Platelet Analysis by Flow Cytometry

To rule out the presence of leukocytes and to demonstrate that platelets had not been activated during the isolation process, 10^6 platelets were stained in 0.5mL JNL buffer containing APC Mouse Anti-Human CD42b (BD Pharmingen), BB515 Mouse Anti-Human CD62P (BD Horizon), and PE Mouse Anti-Human CD45 (BD Pharmingen). Flow cytometry was performed using the FACS Melody (Becton-Dickinson, USA). Analysis was performed using FCSalyzer (Open Source, SourceForge).

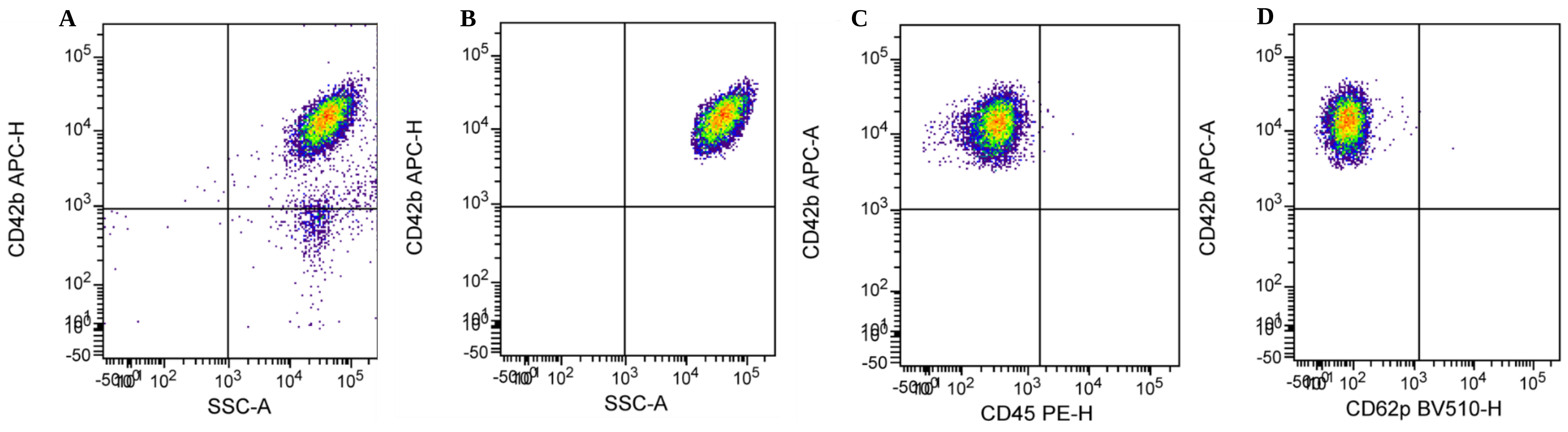

A. Stained platelets are CD42B^{+} ; **B.** Stained platelets gated for CD42B ; **C.** Stained platelets gated for CD42B are CD45^{-} ; **D.** Stained platelets gated for CD42B are CD62P^{-}
