# Supplementary material for: Plasminogen activator inhibitor 1 is associated with high-grade serous ovarian cancer metastasis and is reduced in patients who have received neoadjuvant chemotherapy
Source: Front Cell Dev Biol. 2023 Dec 7;11:1150991. doi: 10.3389/fcell.2023.1150991 (PMC10740207; doi:10.3389/fcell.2023.1150991)
Supplement: Supplementary file 6 [file DataSheet5.PDF]

Additional File 5

Integrative Genomics Viewer

Binary alignment map (BAM) files were converted to bedgraph files and then to bigwig files and visualized in the Broad Institute's Integrative Genomics Viewer, with focus on the SERPIN E1 gene.

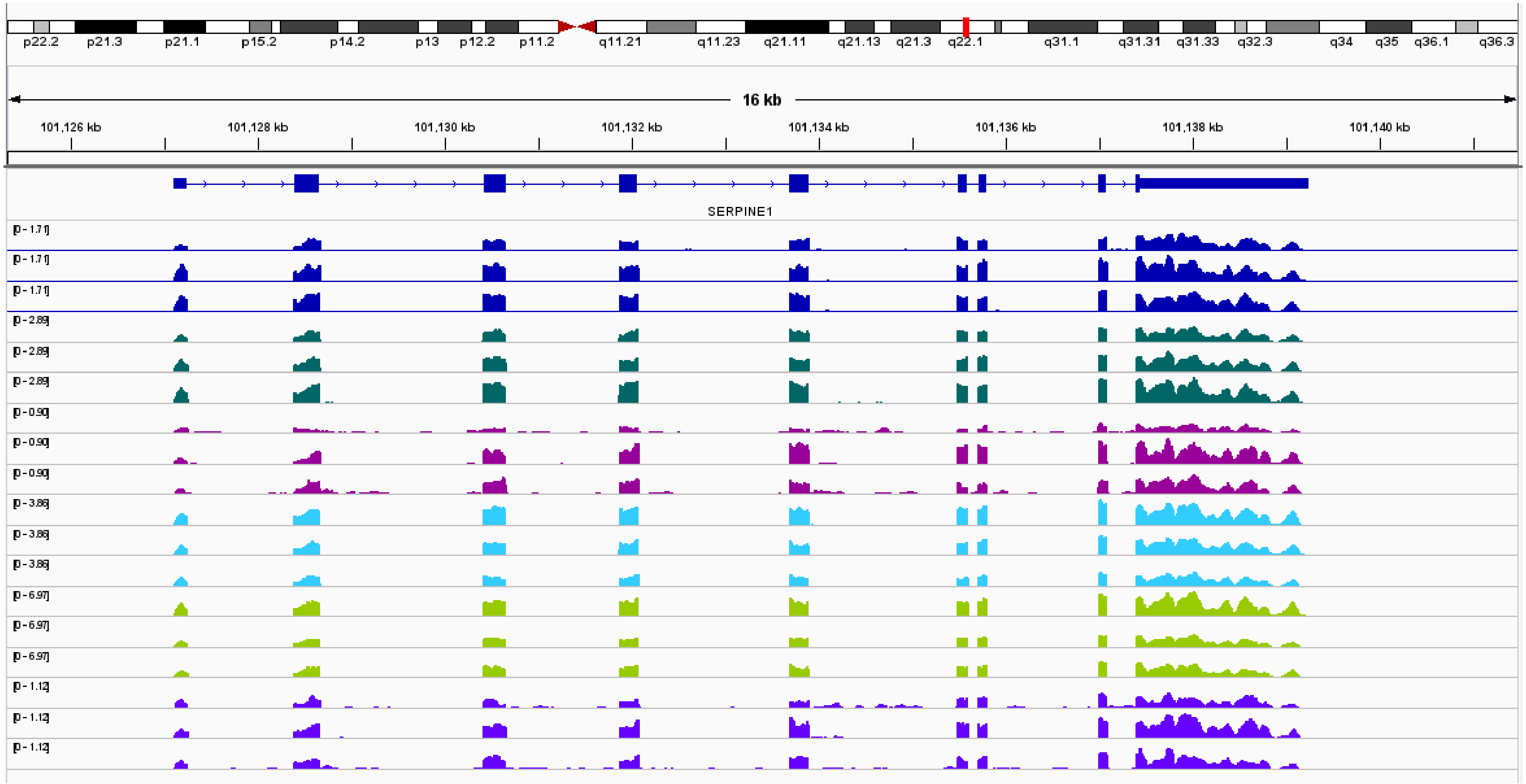

Untreated cells are depicted in dark blue, negative control is dark green, PAI-1 knockdown in pink, untreated cells with platelets in light blue, negative control-treated cells with platelets in light green, and cells treated with PAI-1 siRNA and then platelets in purple.
