# Supplementary material for: Plasminogen activator inhibitor 1 is associated with high-grade serous ovarian cancer metastasis and is reduced in patients who have received neoadjuvant chemotherapy
Source: Front Cell Dev Biol. 2023 Dec 7;11:1150991. doi: 10.3389/fcell.2023.1150991 (PMC10740207; doi:10.3389/fcell.2023.1150991)
Supplement: Supplementary file 7 [file DataSheet8.PDF]

Additional File 8

Preliminary CTC Data TN patients with Stage III/IV HGSOC

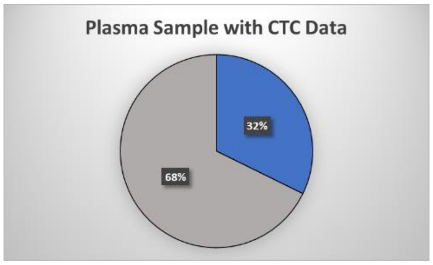

30/93 samples (32.3%) had plasma sample with matched CTC data.

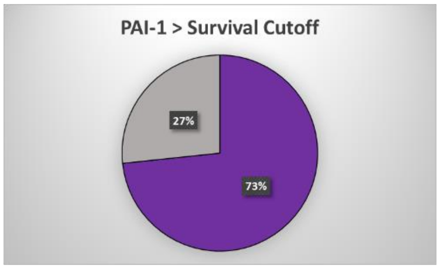

22/30 samples (73.3%) had plasma PAI-1 levels higher than survival cutoff.

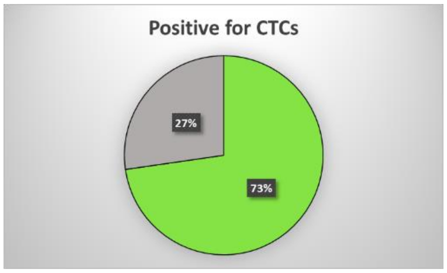

16/22 samples (72.7%) were positive for CTCs.
